# 3D-Cultured Vascular-Like Networks Enable Validation of Vascular Disruption Properties of Drugs *In Vitro*

**DOI:** 10.3389/fbioe.2022.888492

**Published:** 2022-06-13

**Authors:** Prabhusrinivas Yavvari, Anna Laporte, Laura Elomaa, Fabian Schraufstetter, Inga Pacharzina, Aline Dominique Daberkow, Anke Hoppensack, Marie Weinhart

**Affiliations:** ^1^ Institute of Chemistry and Biochemistry, Freie Universität Berlin, Berlin, Germany; ^2^ Institute of Physical Chemistry and Electrochemistry, Leibniz Universität Hannover, Hannover, Germany

**Keywords:** vascular disruption, antiangiogenesis, sandwich assay, lumen, *in vitro* drug testing

## Abstract

Vascular-disrupting agents are an interesting class of anticancer compounds because of their combined mode of action in preventing new blood vessel formation and disruption of already existing vasculature in the immediate microenvironment of solid tumors. The validation of vascular disruption properties of these drugs *in vitro* is rarely addressed due to the lack of proper *in vitro* angiogenesis models comprising mature and long-lived vascular-like networks. We herein report an indirect coculture model of human umbilical vein endothelial cells (HUVECs) and human dermal fibroblasts (HDFs) to form three-dimensional profuse vascular-like networks. HUVECs embedded and sandwiched in the collagen scaffold were cocultured with HDFs located outside the scaffold. The indirect coculture approach with the vascular endothelial growth factor (VEGF) producing HDFs triggered the formation of progressively maturing lumenized vascular-like networks of endothelial cells within less than 7 days, which have proven to be viably maintained in culture beyond day 21. Molecular weight-dependent Texas red-dextran permeability studies indicated high vascular barrier function of the generated networks. Their longevity allowed us to study the dose-dependent response upon treatment with the three known antiangiogenic and/or vascular disrupting agents brivanib, combretastatin A4 phosphate (CA4P), and 6´-sialylgalactose (SG) *via* semi-quantitative brightfield and qualitative confocal laser scanning microscopic (CLSM) image analysis. Compared to the reported data on *in vivo* efficacy of these drugs in terms of antiangiogenic and vascular disrupting effects, we observed similar trends with our 3D model, which are not reflected in conventional *in vitro* angiogenesis assays. High-vascular disruption under continuous treatment of the matured vascular-like network was observed at concentrations ≥3.5 ng·ml^−1^ for CA4P and ≥300 nM for brivanib. In contrast, SG failed to induce any significant vascular disruption *in vitro*. This advanced model of a 3D vascular-like network allows for testing single and combinational antiangiogenic and vascular disrupting effects with optimized dosing and may thus bridge the gap between the *in vitro* and *in vivo* experiments in validating hits from high-throughput screening. Moreover, the physiological 3D environment mimicking *in vitro* assay is not only highly relevant to *in vivo* studies linked to cancer but also to the field of tissue regeneration.

## Introduction

Angiogenesis is the process of new capillary formation out of an existing vasculature by endothelial cells (ECs) in living tissues (Adams and Alitalo, 2007). It is mainly triggered by growth factors that facilitate differentiation and restructuring of polarized endothelial cells in a timed cascade of signaling events to produce a vascular network made of hollow and lumenized capillaries (Carmeliet, 2005). Using these networks, oxygen as well as nutrient transport and supply of the surrounding tissue is ensured. Therefore, solid tumors often exploit angiogenesis from the healthy periphery to cope with their high growth rate-related, excessive supply demands (Bergers and Benjamin, 2003; De Palma et al., 2017). Specially the treatment of late-stage invasive tumors benefits from targeted disruption and degeneration of already established vascular tumor networks compared to conventional antiangiogenic treatment. Classical antiangiogenic compounds target angiogenic signaling pathways such as the vascular endothelial growth factor (VEGF) pathway by either blocking the secreted VEGF or its receptors on the cell surface. Often antiangiogenic, small molecule drugs also alter the existing vasculature and thus exhibit multiple modes of action. Brivanib and its prodrug brivanib alaninate belong to the group of classical antiangiogenic drugs with multiple targets. As an investigational phase III drug, brivanib exhibits antiangiogenic effects *in vitro* and *in vivo* (Siu et al., 2013). Predominantly, tyrosine kinases are inhibited by brivanib blocking VEGF and fibroblast growth factor (FGF) family receptors with IC_50_ values in the nanomolar range (Huynh et al., 2008; Chou and Finn, 2012). In contrast, small-molecule vascular disrupting agents (VDAs) discriminate mature and healthy networks from tumorous capillary networks *via* distinct differences in EC proliferation and permeability (Smolarczyk et al., 2021). Flavonoids such as 5,6-dimethyl-9-oxo-9H-xanthene-4-acetic acid (DMXAA) as potent cytokine inducers or tubulin-binding agents such as combretastatin A4 with its water-soluble prodrug combretastatin A4 disodium phosphate (CA4P) are prominent representatives of VDAs (Hinnen and Eskens, 2007; Porcù et al., 2014). Both drug types increase the permeability of the tumor vasculature resulting in blood leakage, uncontrolled blood coagulation, rapid shutdown of the tumor core from blood supply, and subsequent tumor tissue necrosis (Nagaiah and Remick, 2010; Drzyzga et al., 2021; LINCS, 2022; PubChem, 2022). In clinical trials, a variety of carcinomas have indeed been effectively treated with such antiangiogenic drugs and/or VDAs. However, cancerous cells in the carcinoma rim often survive the VDA treatment and quickly repopulate the tumor. Therefore, simultaneous application of vascular-targeting agents with conventional cytotoxic anticancer therapeutics or sequential combination with radiotherapy to curb tumor proliferation is encountered a promising strategy in the treatment of solid tumors (Siemann and Horsman, 2009; Drzyzga et al., 2021). Current challenges of VDAs in single and combination therapies are adverse effects such as cardiovascular toxicity observed in preclinical and clinical studies, which manifests as cardiac ischemia, hypertension, or atrial fibrillation and requires the establishment of meticulous clinical management protocols (Gill et al., 2019).

When addressing pharmacokinetics, drug metabolism and therapeutic drug effects, *in vivo* animal studies are currently irreplaceable. Yet, advanced *in vitro* models that closely mimic the native vascular network are powerful tools according to the 3R (replace, reduce, and refine) principles with the potential to reduce the number of animal experiments (Langhans, 2018; Weinhart et al., 2019). This is particularly the case, as more reliable estimates of the therapeutic window for subsequent *in vivo* experimentation can be deduced from 3D vs. 2D assays through more refined information on dose-dependent drug efficacies and their respective cytotoxicity profiles. Furthermore, *in vitro* assays generally bear the potential for cost-efficient adaption into a scalable assay platform to screen antiangiogenic compounds in combination with other drugs (Siller et al., 2020; Van Duinen et al., 2020). To our knowledge, there has been no *in vitro* 3D model described to simultaneously validate the antiangiogenic as well as vascular disrupting properties of drugs. In contrast, a myriad of *in vitro* models exists for studying *de novo* angiogenesis processes and to screen antiangiogenic drug properties (Simons et al., 2015). A common method, known as tube formation assay, utilizes the self-assembly potential of ECs grown on the surface of basement membrane extract coatings (DeCicco-Skinner et al., 2014). The formation of chord- or tube-like structures localized in one plane is observed after 2 h with a maximum between 4 and 6 h. These structures resemble vascular networks of early-stage angiogenesis that are short-lived from 24 h to a maximum of 7 days (Kubota et al., 1988) and often lack a proper capillary-like lumen particularly when cultured on laminin-rich matrices (Senger and Davis, 2011). The limited longevity and consequently the inability of such models to mature to complex vascular structures mimicking the (patho)physiology *in vivo* prevents their use for studying long-term drug effects, for example, under repeated dosing or vascular disruption (Brassard-Jollive et al., 2020).

Profuse vascular 3D networks *in vitro* are established when culturing ECs in extracellular matrix mimicking gels such as collagen (Koh et al., 2008), fibrin (Nakatsu and Hughes, 2008), or synthetic hydrogels (Liu et al., 2021) with additional proangiogenic supplements such as phorbol 12-myristate 13-acetate, sphingosine-1-phosphate, and/or angiogenic growth factors such as VEGF. Furthermore, the importance of organo-typic coculture conditions for successful *in vitro* prevascularization has been emphasized (Shafiee et al., 2021). Coculturing ECs with other cells such as mesenchymal stem cells (Uwamori et al., 2017) or human adipose stem cells (hASCs) (Kang et al., 2018; Manikowski et al., 2018; Andrée et al., 2019) within a gel scaffold resulted in the generation of a 3D vascular network that was stable up to day 14.

Though these strategies have demonstrated the importance of the coculture conditions for generating a long-term stable vascular network *in vitro*, they still hold some bottlenecks toward their application for drug screening studies. A) Coculturing ECs with cells of higher metabolic and proliferative rates can cause competition for nutrition and space, thereby interfering with vascular network formation and the balance of matrix degradation and restructuring; B) owing to the presence of a mixed population of cells along with ECs, identifying the individual response of ECs or their morphological changes during vascularization in response to the other cocultured cells is challenging or requires permanent staining (Hetheridge et al., 2011); C) there is still lack of evidence whether the presence of other cells in the vicinity of ECs would have any effect on the structural integrity of the vascular networks formed (Andrée et al., 2019).

Herein, we report the development of an indirect coculture model of human umbilical vein endothelial cells (HUVECs) and human dermal fibroblasts (HDFs), to generate long-lasting, profuse vascular-like networks, while simultaneously overcoming the aforementioned current limitations. Detailed brightfield and confocal microscopy experiments supported by ELISA assays were performed to study the formation and maturation of the vascular-like structures. We present the expedience of our advanced 3D model by studying drug efficacies toward antiangiogenesis and/or vascular disruption with higher accuracy than the still popular tube forming assay.

## Materials and Methods

### Materials

HUVECs and the VascuLife^®^ VEGF Endothelial Medium Complete Kit including basal medium as well as growth factors and supplements (Lifefactors^®^: rh FGF-b [5 ng/ml], ascorbic acid [50 µg/ml], hydrocortisone [1 µg/ml], FBS, L-glutamine [10 mM], rh IGF-1 [15 ng/ml], rh EGF [5 ng/ml], rh VEGF [5 ng/ml], and heparin [0.75 U/ml]) were obtained from CellSystems^®^ (Troisdorf, Germany). Rat collagen type I was supplied from R&D Systems (Minneapolis, United States) under the trade name Cultrex^®^ 3D Culture Matrix. Hoechst 33342, calcein-AM, fluorescein diacetate (FDA), dextran, Texas Red™ (10 and 70 kDa), and the human VEGF/ELISA kit were purchased from Thermo Fisher Scientific (Waltham, MA, United States), propidium iodide (PI), Accutase^®^ (400–600 units/ml), and Dulbecco’s phosphate buffered saline without CaCl_2_ und MgCl_2_ (DPBS) were from Sigma-Aldrich (St. Louis, MO, United States). Albumin fraction V, sodium hydroxide, and Triton X-100 were from Carl Roth (Karlsruhe, Germany) and fetal bovine serum (FBS) from PAN Biowest (Nuaillé, France). High glucose (4.5 g/L) and low glucose (1 g/L) Dulbecco’s modified Eagle’s medium (HG-DMEM and LG-DMEM; 1 mM pyruvate), penicillin/streptomycin, and trypsin 0.5% (10×, W/ EDTA) were ordered from Life Technologies, Gibco (Darmstadt, Germany) and CELLSTAR^®^ 12-well suspension culture plates as well as T-75 cell culture flasks were obtained from Greiner Bio-One (Kremsmünster, Austria) and Costar^®^ Transwell^®^ 12 mm inserts with a 0.4 µm polyethylene terephthalate (PET) membrane from Corning (New York, NY, United States). The human FGF basic ELISA kit (FGF2) was purchased from Abcam (Cambridge, United Kingdom), monoclonal mouse anti-human CD31 antibody from Agilent Technologies (Santa Clara, CA, United States), Alexa Fluor^®^ 488 goat anti-mouse IgG1 antibody from Thermo Fisher Scientific (Waltham, MA, United States), combretastatin A4 phosphate disodium salt from TCI chemicals (Tokyo, Japan), brivanib from Biomol (Hamburg, Germany), and 6'-sialylgalactose sodium salt from Carbosynth (Berkshire, United Kingdom).

### Ethics Statement

HDF isolation from juvenile foreskin subsequent to circumcision (age between 5 and 18 months) and their usage for the studies was following the approval of the Ethics Committee at Charité Universitätsmedizin Berlin, Germany EA1/081/13, after obtaining written informed consent from their parents.

### Cell Culture

HUVECs obtained from Cellsystems^®^ in passage 3 (p3) were thawed and cultured at 5,000 cells∙cm^−2^ in 10 ml Vasculife^®^ Lifefactors^®^ VEGF medium in a T-75 cell culture flask at 37°C in a humidified atmosphere with 5% CO_2_. For further cultivation and expansion, cells were seeded at 5,000 cells∙cm^−2^ per flask (T-75) after enzymatic detachment with Accutase (0.6 ml, 400–600 units∙ml^−1^). Medium exchange was performed every 3 days. For the indirect coculture assay HUVECs in p4-6 were used.

HDFs originating from human foreskin samples were isolated by using a collagenase and dispase protocol as reported previously (Stöbener et al., 2017). Donors were of different ages between 5 and 18 months. Primary fibroblasts were then cultured from p4-7 using HG-DMEM supplemented with 10% FBS at 37°C in a humidified atmosphere with 5% CO_2_. Detachment was performed with 0.05% trypsin. Fibroblasts in p4-8 were used for experiments.

### Indirect Coculture Assay

Fibroblasts were seeded at a density of 12 × 10^3^ cells·cm^−2^ in a 12-well culture plate (∼3.8 cm^2^ surface area) in 1.5 ml LG-DMEM (10% FBS) and cultured for 24 h. Tissue culture treated 12 mm Transwell^®^ inserts with a 0.4 µm pore size were used for the culture of HUVECs inside a collagen gel. Prior to the addition of the gel the inserts were incubated with 1× DPBS for 3 min to wet the surface of the PET membrane. Afterward, a total of 160 µl of collagen solution (type 1 collagen of rat tail origin, 2.5 mg·ml^−1^, details on the preparation can be found in the supporting information (SI)) were added to the inserts (1.1 cm^2^ surface area) in two iterative steps of 100 and 60 µl, respectively, with a 30 min incubation time in between to avoid meniscus formation within the bottom gel layer. Subsequently, the inserts were incubated for 45 min to ensure complete solidification of the gel. HUVECs were then seeded on top of the collagen gel in 500 µl of Vasculife^®^ Lifefactors^®^ VEGF medium at a density of 60 × 10^3^ cells·cm^−2^ and were left to adhere overnight. On the following day, the HUVEC culture medium was gently aspirated, and a second layer of collagen solution (160 μl, 2.5 mg·ml^−1^) was added onto the cells. After 45 min of incubation, the Transwell^®^ inserts were transferred into the wells of the 12-well plate containing a monolayer of fibroblasts with 1.5 ml LG-DMEM (10% FBS). Vasculife^®^ Lifefactors^®^ VEGF medium (500 µl) with a reduced FBS content of 0.5% was added into the Transwell^®^, and this timepoint is referred to as “day 0”. Media exchange with the respective media in both compartments was conducted every 2–3 days. The samples were imaged using a Zeiss Axiovert 200 brightfield microscope equipped with a Zeiss Axiovert camera and a heated stage at regular intervals. For the control condition of HUVEC culture without fibroblasts, the inserts were immersed in 1.5 ml of LG-DMEM in the wells of the 12-well plate only. For studying the VEGF and/or bFGF dependence of vascular network formation, 500 µl of Vasculife^®^ Lifefactors^®^ Medium void of VEGF and/or bFGF (0.5% (v/v) FBS) were used for culture of HUVECs.

### Quantification of Vascular Endothelial Growth Factor and Fibroblast Growth Factor by ELISA

Respective culture supernatants from HDFs and HUVECs were collected before media exchange on day 2, 7, 9, and 14 and were stored at −80°C until further analysis. For ELISA using VEGF Human ELISA Kit (detection limit: 15.6–1,500 pg·ml^−1^, Invitrogen) and Human FGF basic ELISA Kit (FGF2, detection limit: 15.6–1,000 pg·ml^−1^, Abcam), the medium samples were diluted, if required, using the dilution buffer provided by the respective kit (maximum of 1:50) to ensure the concentration of the growth factors to be within the optimal detection range. The assay was performed according to the manufacturer’s protocol.

### Quantification of Vascular-Like Networks

The images obtained from brightfield microscopy of HUVECs were imported to *Image J*, and the number of each type of branching points was manually identified using the cell counter tool. Due to the vast z-extension on the networks, all our attempts to use automated image analysis failed. Two fields of view per technical replicate were evaluated, and the average was calculated for *n* = 3–4. Representative examples of different types of branching points are included in the supplementary material (Supplementary Figure S1). Normalization to achieve the distribution of each type of branching point was carried out using Eq. 1 (where X is the number of branches originating from a branching point, which can be 3, 4, 5, 6, or 7).

$$\text{Distribution of branching points}=\frac{\text{number of branching points of type X}}{\text{total number of branching points}}\cdot 100$$ (1)

The profuseness of the vascular-like networks was determined using the method described by Gondi et al. (2004): The total number of branches was calculated as the sum of branches weighted by the number of branches arising from each branching node, shown by Eq. 2 (*x*_#_ indicates branching point type with # representing the number of branches).

$$\text{Total number of branches}=\;3\cdot x_{3}\;+4\;\cdot x_{4}\;+5\cdot x_{5}\;+6\cdot x_{6}+7\cdot x_{7}\;$$ (2)

### Immunofluorescence Staining

Adapted from the literature (Auler et al., 2017), cells in collagen were fixed with 4% PFA for 2 h before staining. Afterward, the samples were blocked using 5% w/v bovine serum albumin in DPBS for 3 h and subsequently stained with a monoclonal mouse anti-human CD31 primary antibody (1:50) overnight at 4 °C. Following thorough washing with DPBS containing 0.05wt% Tween for 7 h, the primary antibody was detected by incubation with Alexa Fluor^®^ 488 goat anti-mouse IgG1 as a secondary antibody (1:400) overnight. After another washing step using DPBS containing 0.05wt% Tween for 7 h and counterstaining with Hoechst 33342 (1:400), the samples were immersed in mounting medium on custom-made PET slides and imaged *via* confocal laser scanning microscopy (CLSM) using a Zeiss LSM800 confocal microscope.

### Live–Dead Assay and Imaging

To evaluate the viability of cells, a co-staining using FDA (0.46 µM) as a stain for living and PI (16 µM) as a stain for dead cells was conducted. Staining solution (200 µl) (1× DPBS) was added directly onto the collagen scaffolds containing HUVECs and incubated for 8 min at room temperature. Additional Hoechst solution was added to the samples (10 µg·ml^−1^) to counterstain the nuclei wherever required. Subsequently, the solution was removed and the collagen gels were washed thrice with 1x DPBS. The samples were then placed on PET slides for imaging *via* CLSM using a Zeiss LSM 800 confocal microscope. Fifty microliter of 1× DPBS were added onto the collagen gels to prevent drying of the gels during imaging.

### Calcein-AM and Texas Red-Dextran Staining

Collagen gels with HUVEC networks from indirect coculture after day 21 were treated with 1 mg∙ml^−1^ of 10 or 70 kDa Texas red-dextran (TD, dissolved in Vasculife^®^ Lifefactors^®^ medium supplemented with 0.5% FBS) for 24 h and subsequently washed twice with PBS. The samples were then incubated with calcein-AM (10 µM in Vasculife^®^ Lifefactors^®^ medium supplemented with 0.5% FBS) for 2–5 min. Afterward, the samples were washed with PBS, transferred onto PET slides, covered with mounting medium, and imaged *via* CLSM (Zeiss LSM 800 confocal microscope).

### Drug Treatment of the Vascular-Like Networks

The indirect coculture models were prepared and directly incubated inside the Transwell^®^ compartment with different concentrations of investigational drug or prodrug molecules (brivanib (LINCS, 2022)), combretastatin A4 phosphate (CA4P (PubChem, 2022)), or a drug under development (6’-silaylgalactose (SG) (Chung et al., 2019)) in 500 µl Vasculife^®^ Lifefactors^®^ medium (0.5% (v/v) FBS) void of VEGF, either starting from day 0 or day 7 of culture. The drug solutions were replaced on every alternate day with the media exchange. As controls, equal volumes of PBS were added to the HUVECs and for brivanib an additional DMSO vehicle control was conducted. For drug treatment starting from day 7, the fibroblasts in the bottom compartment were replaced 1 day before the commencement of drug treatment. Models were observed for up to 14 days starting from the first administration of the respective drug.

### Statistical Evaluation

Statistical significance of all data was calculated with the software *GraphPad Prism 9.0* (San Diego, CA). The respective number of individual experiments *n* is stated in the figure caption. Error bars indicate standard deviations (SD) in the graphs unless otherwise stated.

## Results and Discussion

### Indirect Coculture for Long-Term Stable Vascular-Like Networks

**FIGURE 1:**
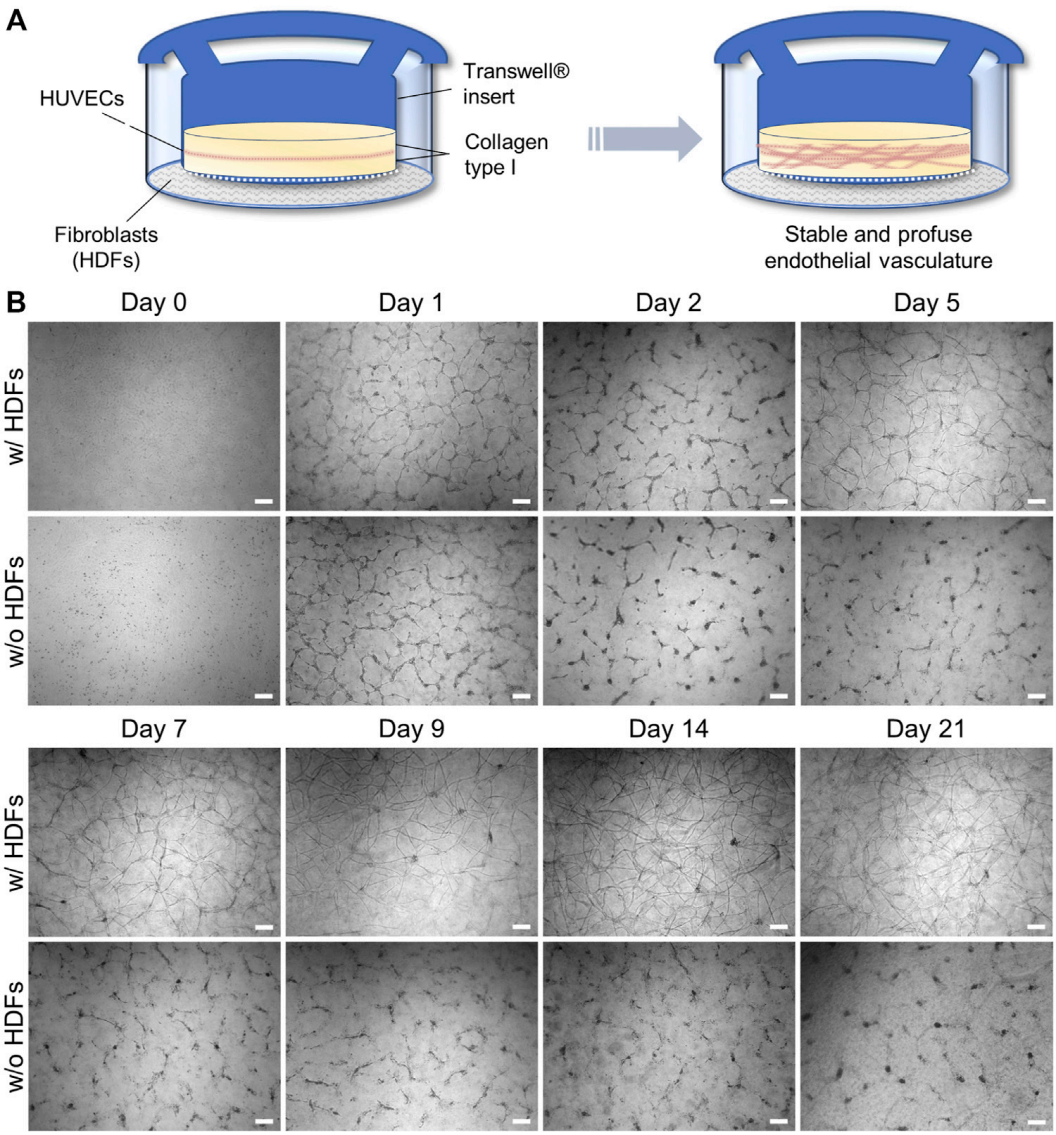
**(A)** Schematic setup of an indirect coculture angiogenesis assay of HUVECs with HDFs to induce 3D vascular-like network formation; **(B)** Representative, time-dependent brightfield microscopy images of the forming HUVEC network cultured with and without fibroblasts. Note that slight meniscus formation at the insert wall during collagen gelation results in a darker contrast at the outer regions of the insert during brightfield imaging (scale bar 200 μm, *n* = 3).

We redesigned an established 3D angiogenesis assay (Montesano et al., 1983) comprising confluent HUVECs sandwiched between layers of rat tail collagen type I to generate a powerful assay setup for long-term drug testing and validation *in vitro*. Transferring the conventional sandwich assay into an insert-culture format according to Figure 1A enables the indirect coculture of HUVECs sandwiched in between two gel layers in the insert with confluent HDFs cultured on the outer well compartment. The key to produce a long-lived vascular-like network *in vitro* in our assay configuration is the indirect coculture with HDFs, which allows potential crosstalk with HUVECs through the membrane of the insert (Figure 1B).

It thus combines the advantages of cell–cell signaling as observed in direct coculture (Montesano et al., 1993; Uwamori et al., 2017; Kang et al., 2018; Manikowski et al., 2018) with the spatial separation of cell types in insert cultures to prevent cellular overgrowth of one cell type. The latter was observed in our initial attempts of a direct coculture of HUVECs and HDFs in a collagen gel (data not shown). During indirect coculture both HUVECs and HDFs were grown in their respective culture media, that is, LG-DMEM supplemented with 10% FBS in the HDF compartment. In the HUVEC compartment VascuLife^®^ VEGF endothelial medium, which is supplemented with recombinant VEGF and basic FGF (bFGF) as important proangiogenic growth factors but with reduced serum concentration (0.5% v/v FBS), was used in order to encourage cellular self-organization over proliferation. The chosen collagen concentration of 2.5 mg·ml^−1^ did not require further stiffness or supplement adjustment of the gel by, for example, blending with agarose or Matrigel^TM^ as reported by Ichanti et al. (2020) to enable efficient EC migration and vascular network formation.

In the absence of HDFs (Figure 1B), the HUVEC capillary structures in this sandwich assay build up through progressive reorganization of the monolayer within 2 days as described in the literature (Montesano et al., 1983; Nakatsu and Hughes, 2008). However, it starts to regress and disintegrate after day 2 when the culture time is extended. Interestingly, in the presence of fibroblasts, the same trend is initially observed, but latest on day 5–7 a full recovery of the vascular-like structures can be detected *via* brightfield microscopy (Figure 1B). These recovered structures appear even more defined than the initial ones on day 1. They form multicellular nodes that give rise to new vascular-like branches or extensions and can viably be maintained beyond day 21. The majority of the vascular network-like structures were developed by day 14, after which the density of the tubular network remained nearly constant, opening the possibility for extended drug testing of, for example, antiangiogenic or vascular disrupting agents under multiple dosing.

**FIGURE 2:**
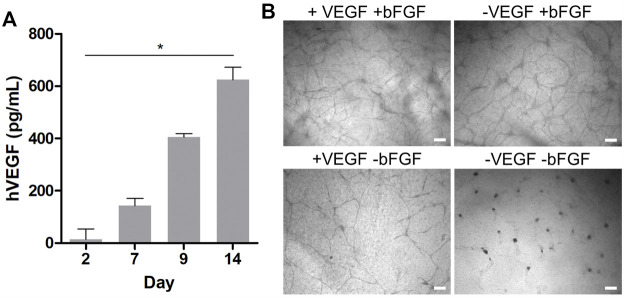
**(A)** Time-dependent quantification of the hVEGF concentration in HDF culture medium *via* ELISA during indirect coculture of HUVECs and HDFs. Data presented as mean ± SD (*n* = 4, *p < 0.05 as determined by Friedman ANOVA); **(B)** brightfield microscopy images of HUVECs grown in indirect coculture on day 14, with and without VEGF and/or bFGF supplementation in the HUVEC medium (scale bar 200 μm, *n* = 3).

The beneficial effect of primary, non-tumor-derived fibroblasts in this setup can be attributed to their potential to produce a myriad of proangiogenic growth factors including members of the VEGF family and bFGF, which synergize in the formation and development of lumenized vascular networks *in vitro* (Newman et al., 2011). To this end, the human VEGF and bFGF content in the HDF and HUVEC compartments was quantified *via* an enzyme-linked immunosorbent assay (ELISA) in a time-dependent manner. Interestingly, no quantifiable amounts of VEGF or bFGF could be detected in the supernatants of the HUVEC compartment at any time starting on day 2, both in the presence or absence of HDFs. However, under indirect coculture, corresponding measurements with supernatants collected from the outer HDF compartment indeed revealed a significant increase of VEGF from 14 to 600 pg·ml^−1^ from day 2 to 14 (Figure 2A). In contrast, no quantifiable amounts of VEGF were found in HDF supernatants from cultures without HUVECs in the insert (data not shown), suggesting that the VEGF production depends mainly on the crosstalk between HDFs and HUVECs in indirect coculture. Our efforts to quantify bFGF by ELISA in the indirect coculture setup in the presence and absence of HUVECs yielded no quantifiable amounts in the supernatants of the fibroblast compartments, although we were able to detect the exogenous bFGF supplement in fresh HUVEC medium (data not shown).

It is well known that unbound growth factors such as VEGF or bFGF are unstable under cell culture conditions with half-lives from 1 to 8 h in the presence of cells (Vempati et al., 2014). At the same time, HUVECs and fibroblasts produce and secrete matrix components such as fibronectin or proteoglycans, which accumulate in the collagen gel and strongly bind and adsorb growth factors. Our results suggest that within 48 h VEGF and bFGF supplemented in the HUVEC culture medium are degraded, consumed, or bound to matrix components in the collagen gel (Griffith et al., 2005). Thus, they might become undetectable in supernatants *via* ELISA. Accordingly, the measurable HDF-derived VEGF concentration in the outer compartment of the assay might reflect only a fractional amount of the VEGF within the gel.

In addition to the effect of HDFs in coculture, we also investigated the effect of the exogenous growth factor supplements in the HUVEC medium on tubular network formation under indirect coculture conditions with HDFs. Therefore, qualitative network assessment was performed in comparison with structures obtained with VEGF and/or bFGF-deficient HUVEC medium in the coculture setup with HDFs. Representative brightfield images on day 14 of the experiments in the presence of HDFs are shown in Figure 2B. While no notable alterations in the formation of HUVEC networks were observed in the presence and absence of VEGF supplement, a clear drop in the network density was observed in bFGF-deficient medium. In the absence of both VEGF and bFGF supplements in the HUVEC medium no network was observable. Because bFGF as a strong proangiogenic inducer is not produced in quantifiable amounts by HDFs in the coculture assay, its supplementation (5 ng·ml^−1^) in the HUVEC medium is essential to arrive at a high network density in this assay. In contrast, supplementation of the HUVEC medium with VEGF (5 ng·ml^−1^) in the presence of HDFs and bFGF supplement is not necessarily required to efficiently generate networks. Notably, the HDF secreted VEGF level quantified in the outer compartment of the indirect coculture assay was eight-fold lower than the exogenous VEGF supplement in the HUVEC medium, but might be much higher within the gel as discussed previously. Nevertheless, the presence of exogenous VEGF does not substitute for HDFs in this assay because no long-lived vascular network formed in the absence of fibroblasts (Figure 1B). Similar observations were made by Newman et al. (2011) with angiogenesis experiments under coculture conditions in fibrin gels. They concluded from their study that a cocktail of proangiogenic factors other than VFGF and bFGF secreted by HDFs is the major trigger of an angiogenic phenotype in endothelial cells, which is consistent with our findings. In the absence of HDFs, exogenous VEGF and bFGF supplementation in HUVEC medium at day 2 appears to be insufficient to further support maturation and stabilization of the generated chord-like structures, leading to their regression and disappearance over time (Figure 1B). However, in the presence of HDFs, the increasingly released HDF-derived VEGF presumably in synergy with other growth factors appears to induce a proangiogenic recovery and further maturation of the tubular network observable latest on day 5–7 in indirect coculture (Figure 1B). Thus, the general VEGF level (5 ng·ml^−1^) in the supplemented HUVEC cell culture medium alone could only initiate the migration and chord-like assembly of HUVECs observed on day 1–2 (with or without coculture) (Figure 1B) but not the subsequent maturation into a 3D vascular-like network. The latter requires the presence of presumably multiple growth factors secreted by fibroblasts.

**FIGURE 3:**
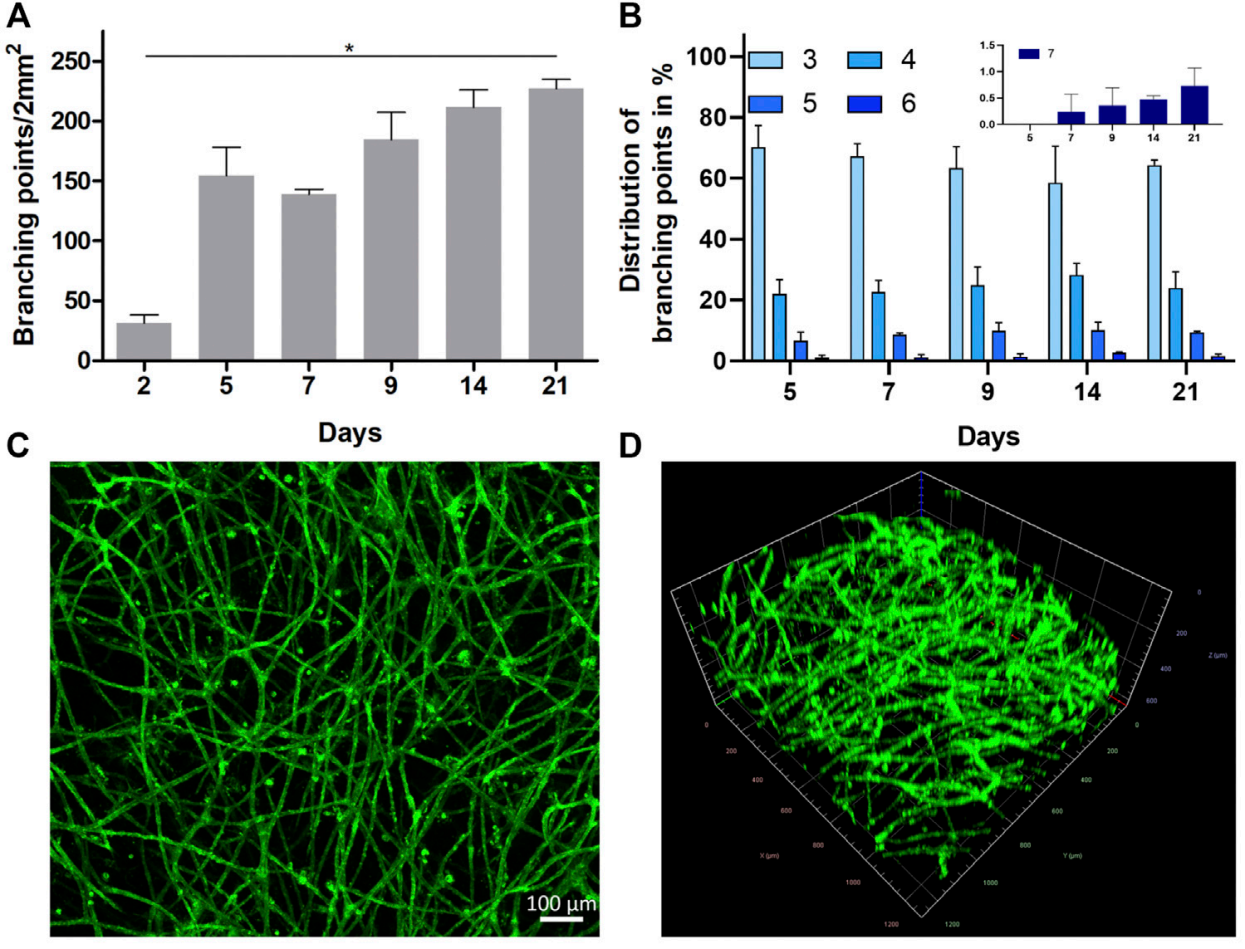
**(A)** Profuseness of the vascular-like network represented as time-dependent total number of branching points per 2 mm^2^ (mean ± SD, *n* = 3, *p < 0.05 as determined by Kruskal–Wallis ANOVA with Dunn’s multiple comparison *post-hoc* test). **(B)** Time-dependent percentage distribution of different branching points in the vascular network (inset shows the zoomed in data of seven branching points for all the groups (mean ± SD, *n* = 4)). **(C,D)** Representative CLSM images (tile stitched) showing the structure of the 3D HUVEC vascular-like network in collagen on day 21 as **(C)** orthogonal projection (scale bar 100 µm, *n* = 4) and **(D)** 3D projection imaged over a z-range of 600 µm after live cell staining with CD31 (1.2 mm^2^).

Potential diffusion-based VEGF gradients from the media compartments toward the initial 2D cell seeding plane within the gel, rationalize the presence of profuse 3D vascular-like networks with a vertical expansion of up to a maximum of 600 µm (Supplementary Figure S2). The profuse nature of the formed 3D vascular-like networks within the gels does not allow automated image analysis, particularly with brightfield images. Therefore, the number of branching points and the number of branches arising from each node (degree of branching) as a function of time was quantitatively accessed from representative images by manual counting (Gondi et al., 2004). In line with visual inspection, the quantitative analysis of the brightfield microscopy images regarding the number of branching points confirms a strong expansion of the vascular-like HUVEC networks from day 2 to 21 (Figure 3A). Analyzing the distribution of the total number of branches arising from each branching point gave more cues on the maturation of the vascular-like networks. In general, we observe mostly nodes with three to five branches between day 5 and 21, while nodes with six branches or more occur less frequent (Figure 3B). The distribution of branching points stays almost constant over the whole assay period. However, latest from day 7 onward it was not possible to get all formed structures in focus during brightfield imaging indicating that the forming vascular-like networks start to grow vertically out of the seeding plane toward the surrounding (Figure 1B). As a result, a highly mature, complex network is observed on day 21 of the culture after CD31 staining (Supplementary Figure S2B, Figure 3C). In addition to straightforward visualization, the CD31-positive network indicates maintenance of the endothelial cell identity during network formation. Furthermore, the presence of CD31 is an essential prerequisite for endothelial cell junctional integrity and thus barrier function (Privratsky and Newman, 2014) similar to vascular endothelial (VE)-cadherin (Gavard, 2014), which was also detectable along the formed vascular-like structures *via* immunohistochemistry (Supplementary Figure S3). From confocal microscopy, the network z-dimensions were determined to be ranging from 100 to 300 µm on average with local extrema up to 600 µm (Figure 3D).

**FIGURE 4:**
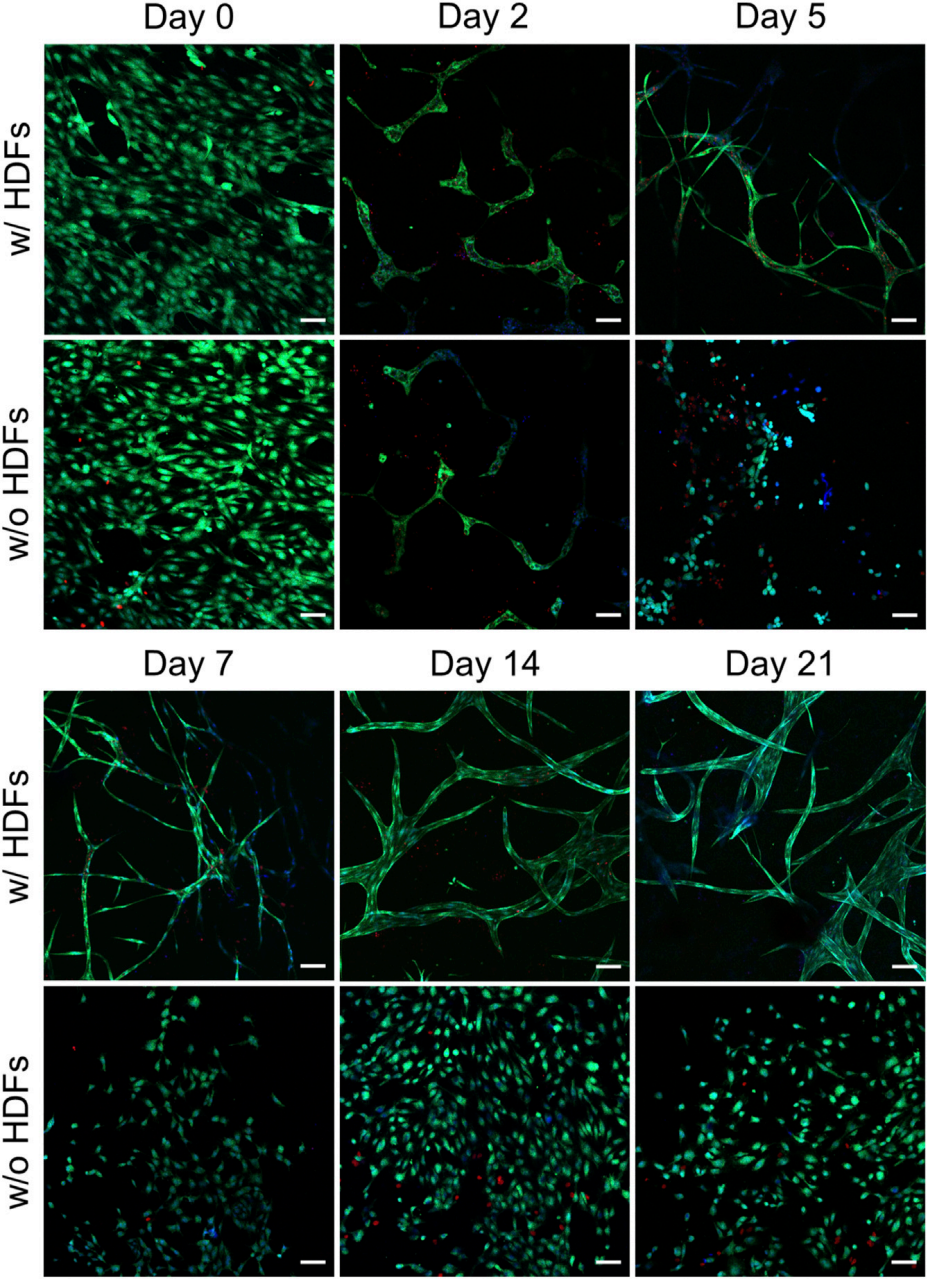
Representative CLSM images from the live–dead assay of HUVECs in collagen gels when cultured with and without fibroblasts (scale bar 100 µm). All images were recorded in a single-confocal plane (day 0, 2, 5, and 7) or within a short z-range of 20 µm (for day 14 and 21). All the images display the merging of Hoechst (blue), FDA (green), and PI (red) channels (*n* = 2).

Although brightfield microscopy indicated the disintegration of the initially formed vascular-like structures in the absence of HDFs, HUVEC viability was not affected over the time course of the experiment. Confocal microscopy from live–dead staining of the samples, both in the presence and absence of fibroblasts, indicated hardly any PI-positive, dead HUVECs over the time course of the experiment (Figure 4). This is in general agreement with findings of direct coculture angiogenesis assays of endothelial cells with mural cells (Evensen et al., 2009). In the absence of HDFs, HUVECs tended to simply proliferate in the initial cell seeding plane after disintegration of the transient chord-like structures on day 2.

In summary, conditions favoring angiogenesis will promote HUVECs to generate capillary networks, whereas confluent cell layers are formed when angiogenesis is discouraged, for example, by a lack of important growth factors. This is in line with the results from previously published literature on direct cocultures of HUVECs with HDFs (Costa-Almeida et al., 2015) or hASCs (Manikowski et al., 2018). However, in our indirect coculture, the induction of capillary-tube formation can be purely assigned to soluble factors from HDFs or molecular crosstalk between HUVECs and the cocultured HDFs, while in direct coculture additional HUVEC-supporting cell–cell contacts or the reorganization of the matrix hydrogels by supporting cells cannot be excluded as mechanistic driving force for tube formation.

### Anastomosing and Lumenized Vascular-Like Networks

**FIGURE 5:**
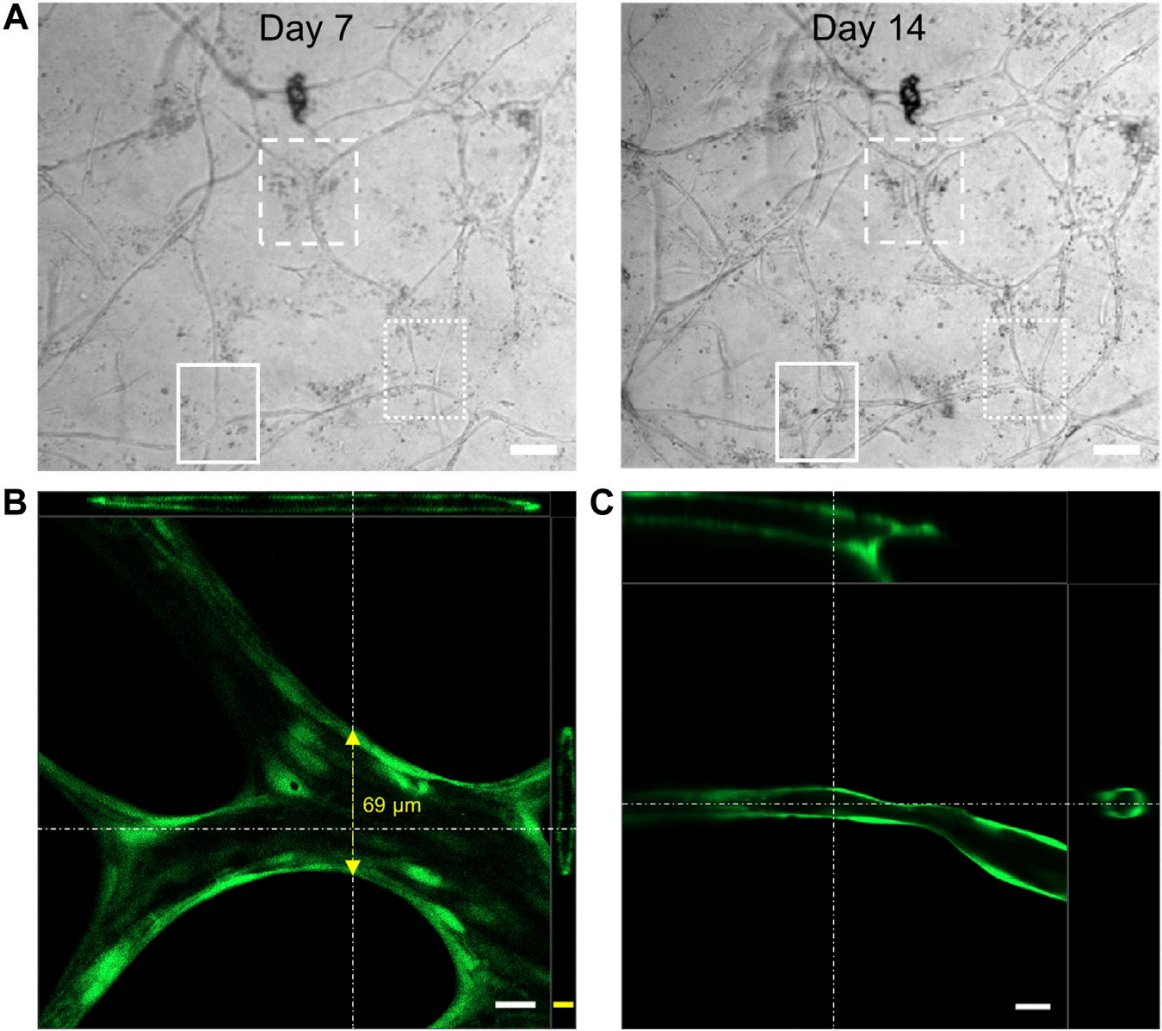
**(A)** Representative brightfield microscopy images of the HUVEC vascular-like network showing anastomosis (highlighted areas) of the cellular extensions giving rise to new junctions (scale bar 100 µm); 3D cross section image generated from confocal microscopy of **(B)** FDA or **(C)** calcein-AM (both green) treated HUVEC network, showing a hollow lumen structure on day 21 (scale bar 20 µm (white), 10 µm (yellow), top-XZ plane projection, right most-YZ plane projection, *n* = 4).

The presence of an active and healthy vascular network *in vivo* is accompanied by anastomosis of newly forming vascular sprouts (Chen et al., 2010; Diaz-Santana et al., 2015) with simultaneous lumen formation (Lammert and Axnick, 2012; Charpentier and Conlon, 2014) improving vascular connectivity and nutrient supply. The indirect coculture strategy and the transparency of the collagen gel matrix allowed us to follow all structural changes of HUVECs during angiogenesis and vascular-like network formation by simple brightfield imaging. The day-wise imaging of the samples at the same positions indicated multiple instances of anastomosis, forming new branching points upon longer experimental time periods (Figure 5A).

Confocal laser scanning microscopy (CLSM) of FDA or calcein-AM stained vascular-like networks indicated the presence of hollow lumenized structures. While the thicker strands exhibited a lumen with a diameter of up to 70 µm (Figure 5B), the thinner vascular structures exhibited a circumferentially uniform lumen of around 10–30 µm (Figure 5C). The lumenized vascular-like networks observed in our studies were in close resemblance to those reported for fibroblast coculture assays by Kim et al. (2013) on a microfluidic chip or by Newman et al. (2011) on beads in a fibrin gel. In conclusion, our current 3D coculture model allows us to form dynamic vascular-like networks that keep growing throughout the experimental time of 21 days with simultaneous lumen formation.

### Early Growing and Mature Vasculature-Like Structures Exhibit Differential Vascular Barrier Function

**FIGURE 6:**
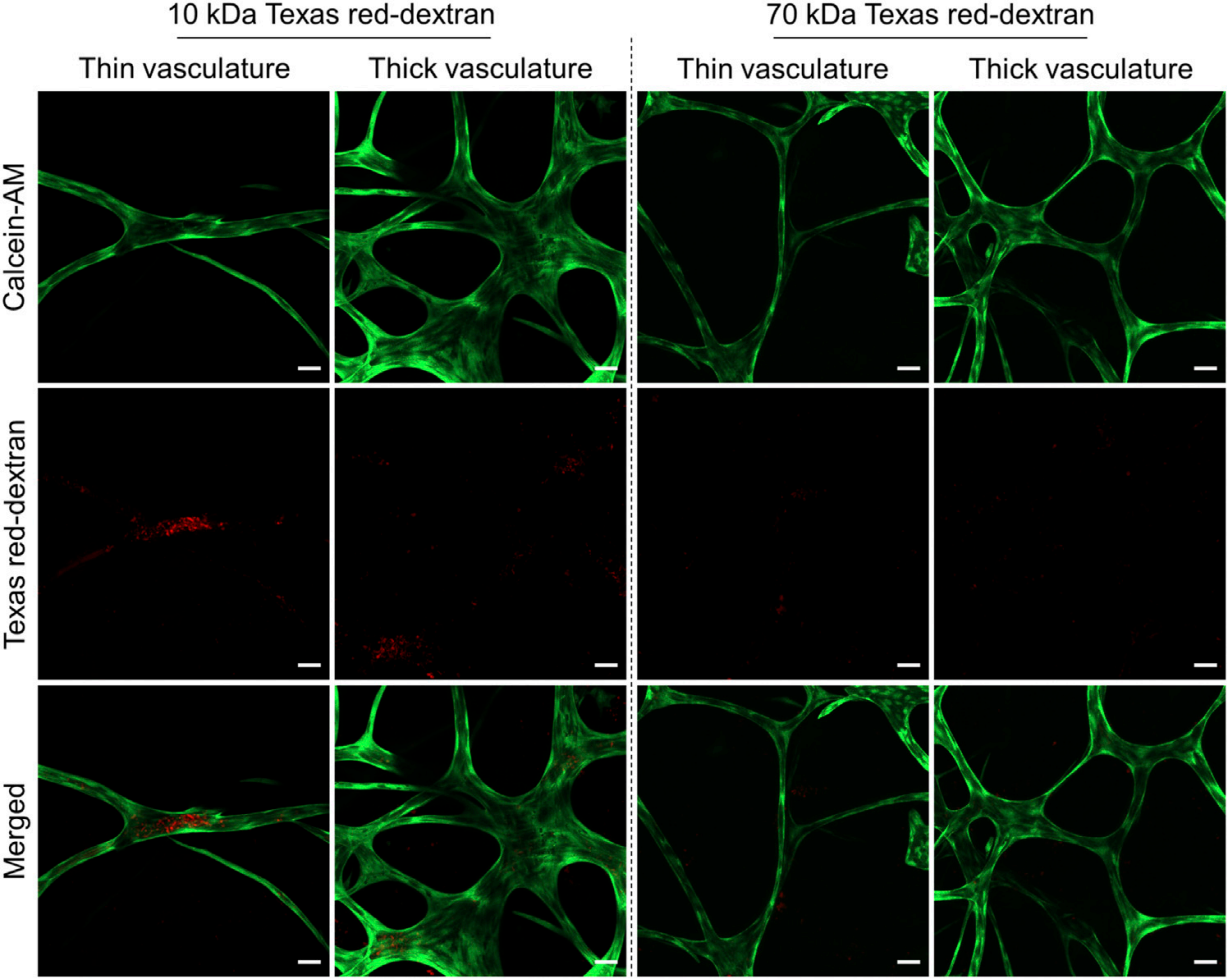
Representative CLSM images showing the permeability of thin and thick branches of the vascular-like networks toward Texas red-dextran (TD, red) of molecular weights of 10 and 70 kDa. HUVECs were stained using calcein-AM (green). All the staining experiments were performed after 21 days of culture (*n* = 4, scale bar 50 µm).

The intercellular adhesive points, known as adherens and tight junctions, between the ECs forming the vascular networks dictate their stability. In addition to influencing the differentiation of the ECs during vascular maturation, mural cells (Kurzen et al., 2002; Alimperti et al., 2017) and endogenously produced proteins and growth factors, such as VEGF (Bates, 2010), also assist in the development and stabilization of stronger intercellular EC contacts increasing the vascular barrier (Jain, 2003). This generally results in lower vascular permeability for endo- and exogenous compounds in blood extravasating the vessel or entering the blood stream from tissue (Heinolainen et al., 2017). To access the structural integrity and the permeability of the vascular-like structures, we employed TD of different molecular weights and detected their uptake into the luminal space after 24 h static TD exposure in the gel (Figure 6). The low-molecular weight dextran (10 kDa) generally permeates endothelial capillaries in both directions *in vitro* and *in vivo*, although the exact mechanism is not known (Egawa et al., 2013; Andrée et al., 2019). In contrast, the 70 kDa TD typically only penetrates EC vascular walls with impaired cell–cell junction integrity and thus lowered vascular barrier function (Alimperti et al., 2017) while it does not penetrate vascular walls reflecting physiological barrier function.

CLSM analysis of the vascular-like 3D-structures exposed to TD for 24 h on day 21 indicated that thin capillaries (3–30 µm diameter) display a higher permeability to 10 kDa TD compared to thicker structures (40–70 µm diameter), while both are impermeable to 70 kDa TD (Figure 6). Thus, we attribute features of immature newly forming or early vascular strands to the thinner vascular structures while the thicker diameter strands reflect more mature vasculature characteristics. Compared to the direct cocultures of green-fluorescent protein (GFP)-expressing HUVECs and hASCs (Andrée et al., 2019), the amount of internalized 10 kDa TD observed in our study is low and was most prominent only at a few sites of early vascular-like structures. Control experiments with GFP-HUVECs under our indirect coculture conditions indicate a lower barrier function of GFP-HUVECs than non-transfected HUVECs based on the amount of accumulated TD inside the lumen (Supplementary Figure S4). Hence, it appears that the indirect coculture of HDFs and HUVECs results in more stable and less permeable vascular-like networks. In addition to adherens (Supplementary Figure S3) and tight junction formation, in general the luminally located endothelial glycocalyx contributes significantly to the barrier function of the capillary wall and will as such be addressed in future studies.

### Blocking the Vascular Endothelial Growth Factor Signaling in Human Umbilical Vein Endothelial Cells Prevents Vascular Network Formation

Since the vascular network formation in our setup is highly dependent on HDF-produced proteins and growth factors such as VEGF, we evaluated the HUVEC network formation under indirect coculture conditions in the presence of a VEGF receptor 2 signaling inhibitor in the HUVEC medium compartment. Therefore, brivanib as effective VFGF and bFGF receptor inhibitor was used as the model drug (Chou and Finn, 2012). Although the route of drug exposure in our model does not exactly mimic the intravenous route of administration, the observed effects may mimic the consequences after intratumoral and intraperitoneal injections or drug extravasation from leaky blood vessels at the tumor site. In all these scenarios, the drug acts directly on the outer surface of the vascular networks.

**FIGURE 7:**
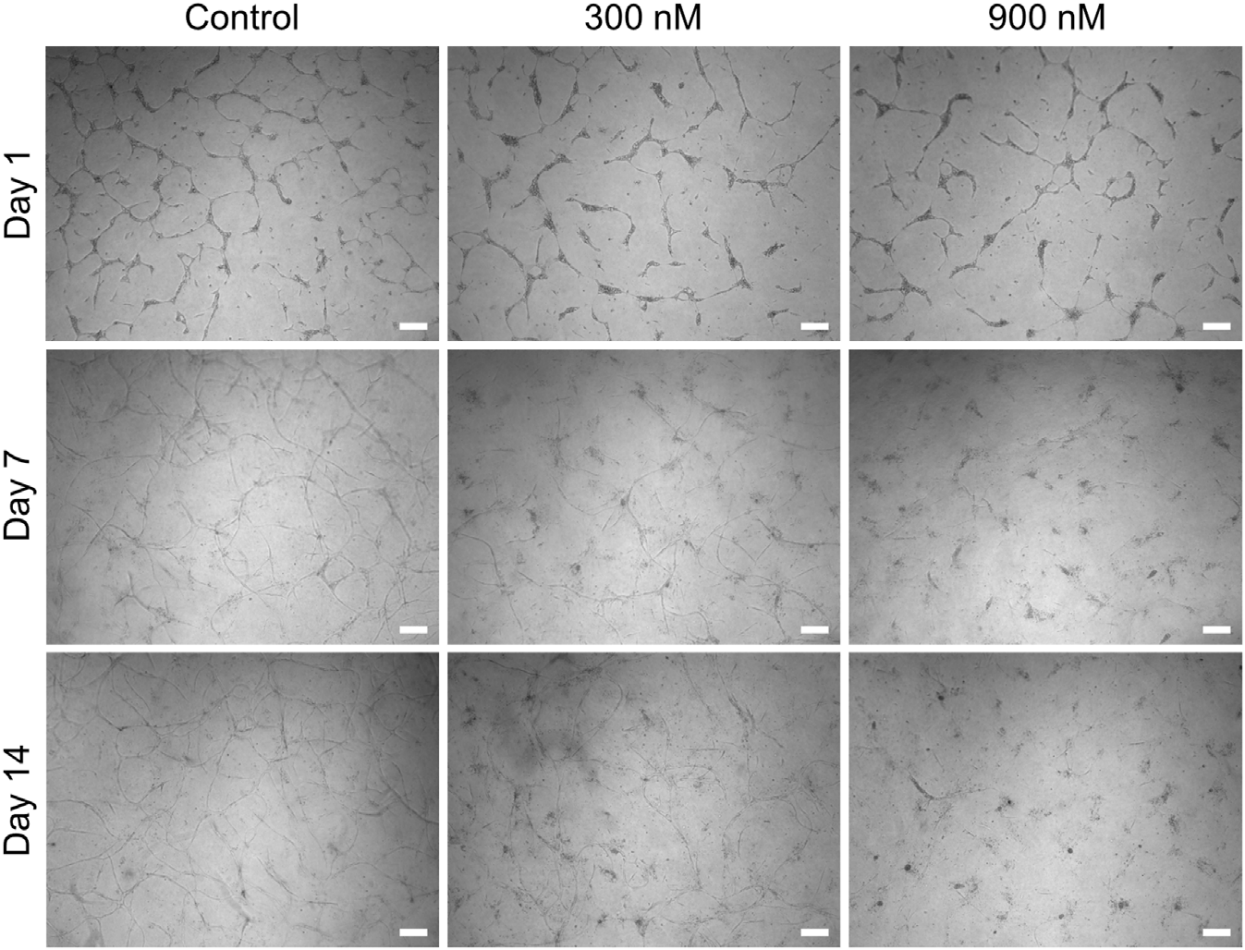
Representative brightfield images showing the concentration-dependent effect of brivanib (0, 300, and 900 nM) on HUVEC network formation in the indirect coculture model (scale bar 200 µm, *n* = 3).

Continuous treatment of the indirect coculture with brivanib starting on day 0 was accomplished through bolus administration (300 and 900 nM) to the HUVEC medium in the upper compartment, which was repeated every other day simultaneous to the medium exchange. A concentration-dependent efficacy toward the inhibition of angiogenesis was observed by a marked reduction in continuous vascular-like networks resulting in total suppression on day 14 with 900 nM brivanib (Figure 7). In addition, the indirect coculture model was treated with an equivalent amount of DMSO as the vehicle control for the respective brivanib doses with subsequent live–dead staining of the cultures. No regression of angiogenesis or dead cells were observed in either of the vehicle controls (Supplementary Figure S5A). Interestingly, we also have found that withdrawal of brivanib treatment in the indirect coculture setting on day 14 allowed the HUVECs to proliferate and recover the vascular-like networks within the following 14 days (Supplementary Figure S5B), clearly indicating the reversible effect of brivanib on capillary formation in this assay setup by targeting the VEGF/VEGF receptor 2 axis. This reversibility of the vascular-like network generation reflected by our model makes it a valuable tool, especially when accessing optimized timepoints for repeated drug dosing. Motivated by the response of this advanced model toward drug treatment and the differential permeability of the early and mature vascular-like networks, we further investigated the effect of other known vascular targeting drugs such as VDAs.

### Validation of Vascular Disruption Properties of Drugs on Vascular-Like Networks From the 3D Culture Model

**FIGURE 8:**
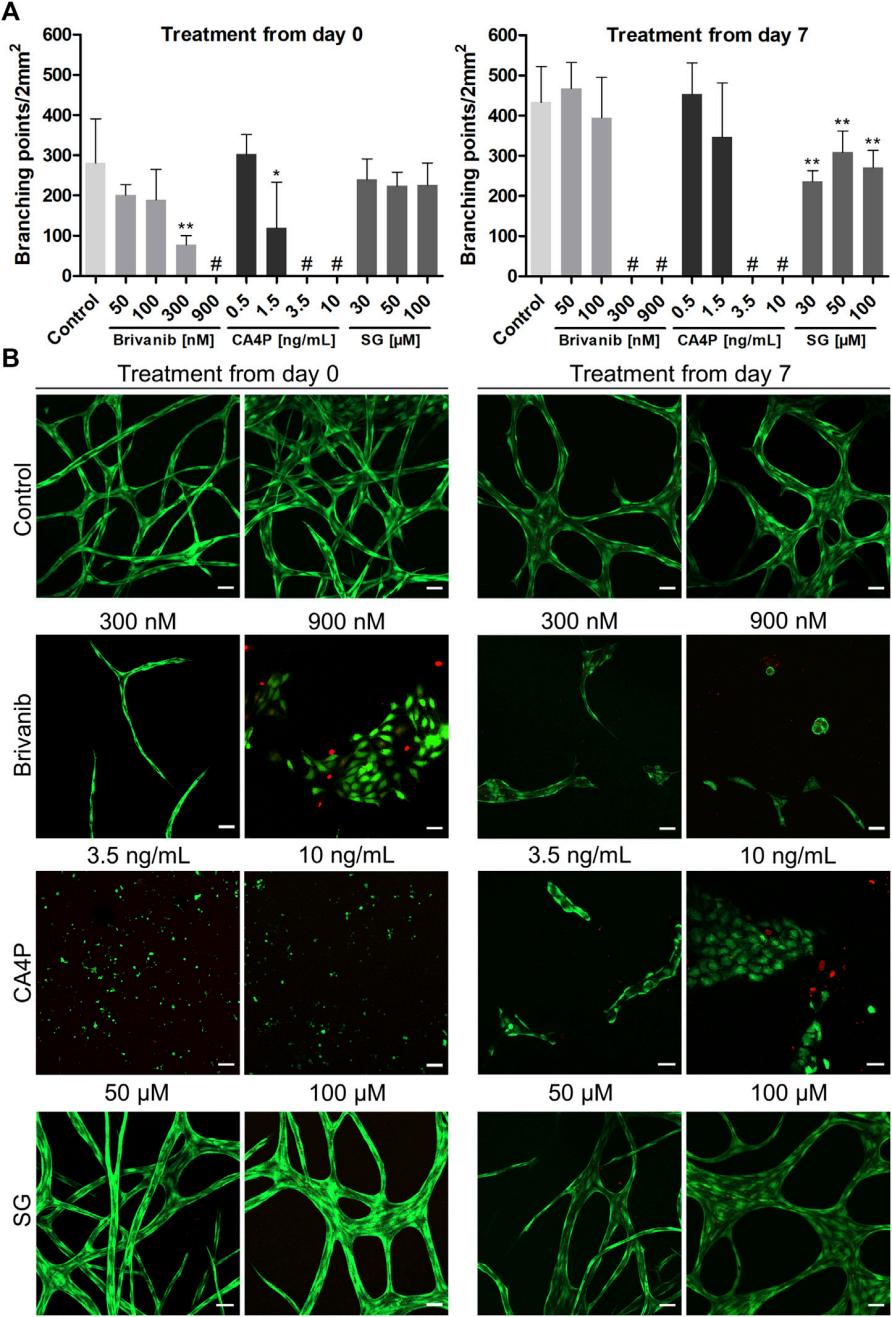
Influence of concentration-dependent drug treatment with brivanib, CA4P, and SG toward angiogenesis (treatment from day 0 onward) and vascular network disruption (treatment starting from day 7) for 14 days, respectively, in indirect coculture of HUVECs and HDFs. **(A)** Quantitative analysis of the respective vascular networks’ number of branching points per area at the end point of the drug treatment (# indicates lack of any quantifiable continuous vascular networks) (mean ± SD, *n* = 5, *p < 0.05, and **p < 0.01 value is significantly different from control as tested by a non-parametric Mann–Whitney U-Test); **(B)** Representative CLSM images of live–dead stained samples [FDA (green), PI (red)] after concentration-dependent treatment with brivanib, CA4P, or SG, respectively, compared to controls without treatment on day 14 and 21 (scale bar 50 µm, *n* = 5).

In addition to brivanib, we chose combretastatin A4 phosphate (CA4P), an investigational drug belonging to the group of tubulin-binding VDAs (West and Price, 2004), and 6’-silaylgalactose (SG), a recent experimental molecule targeting the VEGF receptor 2 on endothelial cells (Chung et al., 2019), to further challenge and validate our model. CA4P induces microtubule destabilization by ß-tubulin binding in endothelial cells which in turn reduces the stability of vascular networks. Simultaneous impairment of the functional engagement of the endothelial cell-specific junctional molecule VE-cadherin hinders stabile neovessel formation, resulting in both antiangiogenic and vascular disrupting effects *in vivo* (Vincent et al., 2005). The antiangiogenic property of the synthetic disaccharide SG was reported *in vivo* while its vascular disrupting properties have not been studied yet (Chung et al., 2019). Thus, we tested for the antiangiogenic effect of the drugs by starting the treatment on day 0 of the indirect coculture as described for brivanib above. Additionally, the evaluation of their vascular disrupting properties on already established vascular-like structures was accomplished by starting the drug treatment on day 7 of the coculture experiment, which was continued until day 21. In both cases, the drugs were administered as bolus to the HUVEC compartment simultaneous to the media exchange which was repeated every other day for 14 days. The combined quantitative analysis of the number of network branching points per area in brightfield images from concentration-dependent brivanib, CA4P and SG treatments starting on day 0 and day 7 are graphically illustrated in Figure 8A. Rational drug concentrations were deduced from *in vitro* data on the tube formation assays with endothelial cells on Matrigel^TM^ reported in literature which locate in the medium nM to low µM-range for brivanib (Li et al., 2020), the low nM-range for CA4P (Vincent et al., 2005) and the low µM-range for SG (Chung et al., 2019). Based on the metabolic activity of viable HUVECs and HDFs in the presence and absence of the drugs, their concentration-dependent cytocompatibility was accessed with an MTS proliferation assay (Supplementary Figure S6). Therefore, cells were seeded on TCPS at seeding densities resembling the ones in the indirect coculture setup. Cell viabilities of drug treated HDFs and HUVECs were not significantly different from controls at all tested concentrations. Only with CA4P a slightly reduced metabolic activity of HUVECs was observed at the highest dose of 3.5 and 10 ng∙ml^−1^ (Supplementary Figure S6A).

At the end of 14-day drug treatments, all samples were imaged *via* CLSM after a live–dead staining to qualitatively access the network structure and cell viability (Figure 8B). The concentration-dependent antiangiogenic efficacy of brivanib indicated in brightfield and CLSM images is in general agreement and is markedly visible by a significantly reduced network integrity at concentrations ≥300 nM brivanib, while full inhibition of angiogenesis is observed at 900 nM brivanib. Brightfield images at lower concentrations of 100 and 50 nM brivanib (Supplementary Figure S7) show a slightly reduced network density at 100 nM compared to 50 nM brivanib treatment and control. Furthermore, brivanib was also able to induce significant vascular disruption of existing vasculature starting at concentrations between 100 and 300 nM during a 14-day treatment. At 900 nM complete disruption was observed (Figure 8; Supplementary Figure S8).

Similarly, CA4P turned out to be very effective in preventing angiogenesis as well as inducing vascular disruption in a 14-day treatment at concentrations ≥3.5 ng·ml^−1^ (Figure 8; Supplementary Figures S9, S10). Closer evaluation of different z-layers in the live–dead stained samples revealed a toxic effect of the drug on HUVECs at the high concentration of 10 ng·ml^−1^ (Supplementary Figure S11), which was more pronounced when treatment started on day 0 of the indirect coculture. This could be an effect of prolonged exposure to the drug and was similarly observed in other *in vitro* studies showing that CA4P is cytotoxic for proliferating but not for quiescent HUVECs (Dark et al., 1997). For brivanib-treated cells, evaluation of different z-planes revealed a lower cell number for 900 nM starting from day 0 and already for 300 nM starting from day 7 (Supplementary Figure S11). This effect was also observed in *in vitro* studies showing the VEGF- and bFGF-stimulated HUVEC proliferation to be reduced in the presence of brivanib (Bhide et al., 2006). In addition, a distinct morphological change with reduced cell surface of the HUVECs was observed after treatment (Supplementary Figure S11), most prominent with CA4P, but also for brivanib treatment with 900 nM starting from day 7, which is for CA4P well described in literature and concomitantly observed with cytoskeleton alterations finally leading to cell apoptosis (Galbraith et al., 2001; Kim et al., 2012).

In the case of SG treatment brightfield microscopy and CLSM images indicated a clearly less pronounced antiangiogenic and/or vascular disrupting effect if compared to the other two tested drugs (Figure 8; Supplementary Figures S12, S13). For treatment starting from day 7, a significantly reduced number of branching points was visible for all tested concentrations if compared to controls but the effect was nowhere near a complete vascular disruption (Figure 8). At 100 µM concentration, additionally, a minor impact on the network morphology was observable for treatments starting on day 0 and 7 with thicker junctions (Supplementary Figures S12, S13). Moreover, with all concentrations of SG tested, no cytotoxicity for the vascular-like network was observed (Figure 8B), which is in line with HUVEC proliferation assays reported in the literature (Chung et al., 2019) or MTS data shown in Supplementary Figure S6.

For direct comparison with the effective drug concentrations identified in our indirect coculture assay, we additionally performed a conventional 24h-tube forming Matrigel^TM^ assay with bolus administration of the drugs along with HUVEC medium (Supplementary Figure S14) similar to previous reports (Vincent et al., 2005; Chung et al., 2019; Li et al., 2020). We found brivanib and CA4P to completely inhibit angiogenesis at concentrations as low as 50 nM and 0.3 ng·ml^−1^, respectively, and largely inhibited angiogenesis at 10 µM SG (Supplementary Figure S14). Thus, the inhibitory concentrations obtained from this tube formation assay are clearly overestimating the efficacy of the drugs compared to our long-term 3D assay in indirect coculture, even when considering the slightly higher cell numbers used in the indirect coculture compared to the tube formation assay. The pure 3D collagen type I matrix acts as an additional extracellular barrier to the drug and resembles the interstitial matrix of tumors in terms of permeability for small molecules (Ramanujan et al., 2002).

Expectedly, CA4P showed the strongest antiangiogenic and vascular disruption effect of all three tested drugs with efficacies already in the low nanomolar range, followed by brivanib while SG displayed only a minor effect. Neither prevention of angiogenesis nor effective vascular disruption was observed in the 3D environment of our advanced model at the highest tested SG concentration of 100 µM. This is in general agreement with the relatively high SG concentration (30 µM) required to inhibit VEGF-induced VEGF receptor 2 phosphorylation in HUVECs (Chung et al., 2019). Although reported as effective antiangiogenic agent in various mouse models (Chung et al., 2019), including a model of pathologic angiogenesis in the mouse retina, its potential as a single-antitumor drug is debatable as SG, based on our experiments, does not benefit from a strong combined antiangiogenic and vascular disruption effect *in vitro*.

Throughout the drug treatment experiment, the confluent HDF monolayer in the lower compartment of the assay was retained without any obvious cytotoxic effects of the drug as detected by brightfield microscopy and MTS viability assays (Supplementary Figures S15, S16). This reinstates the advantage of the indirect coculture enabling to study the behavior of HUVECs toward drug treatment selectively and independent of neighboring cocultured mural cells. Since both HUVECs and HDFs generally transit to a quiescent state once the vascular structures or the confluent monolayer have formed, they are less prone to cytotoxic effects of the drugs, particularly with CA4P (Dark et al., 1997). Therefore, the option to screen drug effects on an already established vascular-like network of quiescent endothelial cells combines with reduced susceptibility of the network for cytotoxic drug effects in this advanced assay.

## Conclusion

In summary, we have established an indirect coculture setup to generate long-lasting, profuse HUVEC-based vascular-like networks in type I rat collagen gels that feature permeability characteristics of mature as well as early vasculature with tubular branches capable to anastomose *in vitro*. The HDF-produced soluble proangiogenic and network stabilizing factors such as VEGF in this system enabled the continuous growth and maturation of lumenized vascular-like networks throughout the whole experimentation period up to day 21 and beyond. Unlike conventional *in vitro* angiogenesis assays that are typically short-lived, the unique combination of long lasting, profuse networks offered by our 3D model allows us to directly image morphological changes in the HUVEC-only network, offering advantages in analyzing drug efficacy with prolonged drug administration up to 14 days. Importantly, the model enables the study of both antiangiogenic and vascular disrupting drug efficacies. The drug effects are selectively acting on ECs without interference of surrounding mural cells, which can otherwise compete for nutrients or space. These truly 3D vascular-like networks expanding up to 600 µm in z-direction attenuated the antiangiogenic drug effects of the model drugs brivanib, CA4P, and SG compared to conventional tube forming assays. The closer resemblance of *in vivo* conditions by the 3D features including the extracellular matrix barrier, the unique possibility to study the antiangiogenic and disrupting drug effects on both early and mature vascular-like structures as well as their longevity highlight the superiority of this advanced model over current state-of-the-art *in vitro* angiogenesis models. In an upcoming study the perfusion of such tubular structures will be addressed. With the established model, particularly information on repeated, prolonged drug administration effects become accessible. Therefore, we envision this advanced model as a predictive tool when validating hits from prescreenings of vascular targeting therapeutics *via* conventional, fast angiogenesis assays, thus bridging the gap between *in vitro* and *in vivo* studies.

## Data Availability

The datasets presented in this article will be made available by the first author upon reasonable request. Requests to access the datasets should be directed to PV, prabhusrinivas.y@gmail.com.
