# Supplementary material for: 3D-Cultured Vascular-Like Networks Enable Validation of Vascular Disruption Properties of Drugs In Vitro
Source: Front Bioeng Biotechnol. 2022 Jun 13;10:888492. doi: 10.3389/fbioe.2022.888492 (PMC9234334; doi:10.3389/fbioe.2022.888492)
Supplement: Supplementary file 1 [file DataSheet1.PDF]

Supplementary Material – Yavvari et al. 2022

Outline

1. Materials and Methods 2
    1.1 Materials 2
    1.2 Formation of collagen gel 2
    1.3 Immunofluorescence staining 2
    1.4 TD permeability with GFP-HUVEC network 2
    1.5 MTS assay 3
    1.6 Matrigel™ assay 3
2. Suppl. Figure 1. BFMS images indicating the various types of branching points 4
3. Suppl. Figure 2. CFMS image of the whole scaffold with vascular-like networks 5
4. Suppl. Figure 3. CLSM images of VE-cadherin stained vascular-like network 6
5. Suppl. Figure 4. CLSM images showing permeability of GFP-HUVEC networks 6
6. Suppl. Figure 5. DMSO vehicle control and retrieval of brivanib treatment 7
7. Suppl. Figure 6. MTS cell viability assay of HDFs and HUVECs treated with drugs 8
8. Suppl. Figure 7. BFMS images of brivanib treatment starting on day 0 9
9. Suppl. Figure 8. BFMS images of brivanib treatment starting on day 7 10
10. Suppl. Figure 9. BFMS images of CA4P treatment starting on day 0 11
11. Suppl. Figure 10. BFMS images of CA4P treatment starting on day 7 12
12. Suppl. Figure 11. BFMS images of live-dead stained samples after drug treatment 13
13. Suppl. Figure 12. BFMS images of SG treatment starting on day 0 14
14. Suppl. Figure 13. BFMS images of SG treatment starting on day 7 15
15. Suppl. Figure 14. Matrigel™ tube formation assay with HUVECs 16
16. Suppl. Figure 15. BFMS images of the HDFs in coculture post drug treatment 17
17. Suppl. Figure 16. MTS assay of HDFs post drug treatment 18

1 Materials and methods

1.1 Materials

Rat collagen type I (# 3447-020-01) was supplied from R&D Systems (Minneapolis, USA) under the trade name Cultrex® 3-D culture matrix. Hoechst 33342 was purchased from Thermo Fisher Scientific (Waltham, MA/USA). GFP-labeled HUVECs were purchased from Cellworks (Caltag Medsystem Company, Buckingham, UK). MTS (ab223881) was purchased from Abcam (Cambridge, UK). Corning™ Matrigel™ was obtained from Thermo Fisher Scientific. Monoclonal mouse IgG2a anti-human VE-cadherin antibody was purchased from Santa Cruz Biotechnologies (USA) and ibiTreat μ-dishes from ibidi GmbH (Gräfelfing, Germany).

1.2 Formation of collagen gel

3D Culture Matrix Rat Collagen I (5 $mg \cdot mL^{-1}$) was used to form the 3D matrix of the indirect coculture assay. For the manual casting of the gel layers a collagen solution with a concentration of 2.5 $mg \cdot mL^{-1}$ was created by the following recipe: 500 µL of collagen solution were mixed iteratively with sterile 10x DPBS (100 µL), 0.1 M NaOH (110 µL) and cell culture grade H_2O (290 µL) on ice. The prepared collagen solution was immediately used within 5 min for setting up the indirect coculture assay, because the pH raise directly initiates gelation and solidification. This process is completed after incubation for 45 min at 37 °C.

1.3 Immunofluorescence staining

Cells in collagen were fixed with 4 % PFA for 2 h before staining as described in the main manuscript. Afterwards, samples were blocked using 5% w/v bovine serum albumin in DPBS for 3 h and subsequently stained with a monoclonal mouse anti-human VE-cadherin primary antibody (1:100) overnight at 4 °C. Following thorough washing with DPBS containing 0.05 wt% Tween for 7 h, the primary antibody was detected by incubation with Alexa Fluor® 488 goat anti-mouse IgG1 as a secondary antibody (1:400) overnight. After repeating the thorough wash and counterstaining with Hoechst 33342 (1:400), samples were immersed in mounting medium on an ibiTreat μ-dish (35 mm) and imaged using Zeiss LSM800 confocal microscope.

1.4 TD permeability with GFP-HUVEC network

Collagen gels with GFP-HUVEC networks from indirect coculture after day 21 were treated with 1 $mg \cdot mL^{-1}$ of 10 kDa Texas red-dextran (TD, dissolved in Vasculife® Lifefactors® medium

supplemented with 0.5% FBS) for 24 h and subsequently washed twice with PBS. The samples were then transferred onto PET slides, covered with mounting medium and imaged using Zeiss LSM800 confocal microscope.

1.5 MTS assay

HDFs or HUVECs were seeded in a 12-well plate (18,500 cells cm^{-2}) and left to adhere overnight. Afterwards, the culture medium was replaced with medium containing the respective drug concentrations and cells were incubated for 48 h. Subsequently, the medium was replaced with fresh medium containing MTS solution (0.33 $mg{\cdot}mL^{-1}$) and the cells were incubated further for 1.5 h. Thereafter, the medium was collected and the respective absorbance was measured at 490 nm. The relative viability was calculated by normalization to the absorbance of the control samples.

1.6 Matrigel™ assay

The bottom surface of a 12-well plate was coated with 120 µL of Matrigel™ and incubated at 37 °C for 20 min. HUVECs were then seeded onto the coated surfaces (18,500 $cells{\cdot}cm^{-2}$) in 1 mL Vasculife® Lifefactors® VEGF medium. For the drug treatment, medium containing the respective drug concentration was added onto the cells. They were then incubated for 24 h and subsequently imaged using brightfield microscopy to detect the formation of vascular tubules.

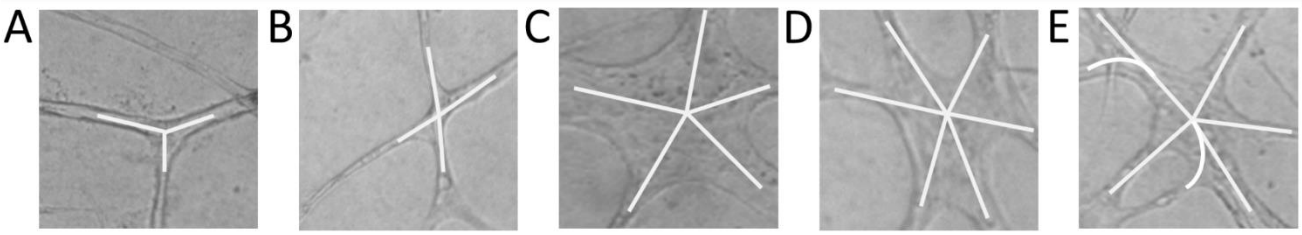

Supplementary Figure 1. Representative brightfield images indicating the various types of branching points: **(A)** 3 branches, **(B)** 4 branches, **(C)** 5 branches, **(D)** 6 branches, **(E)** 7 branches originating from a central node. These branching points were used for the quantification of the vascular network profuseness in indirect coculture of HUVECs and HDFs.

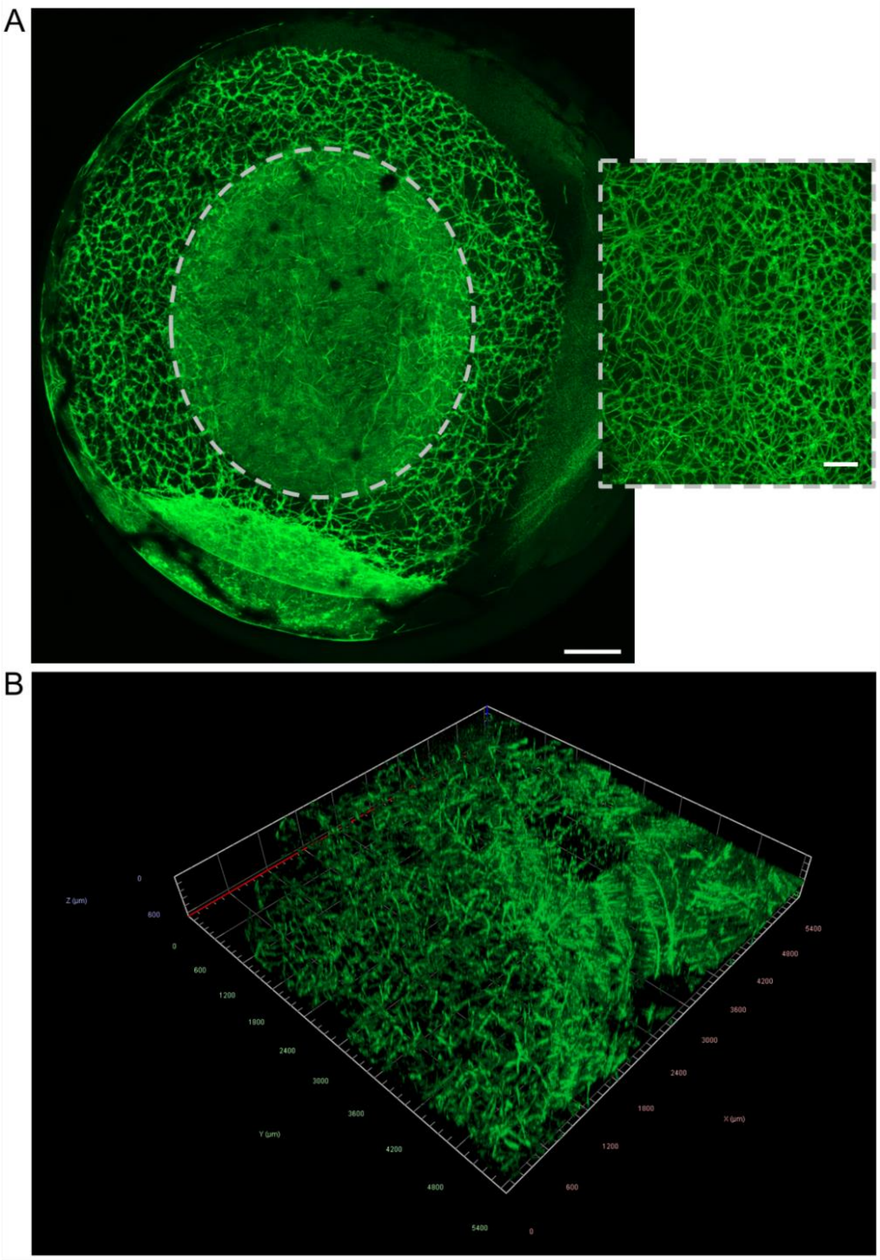

Supplementary Figure 2. Tiled stitched confocal microscopy image of **(A)** the whole gel scaffold (scale bar 1000 µm) indicating the formation of profuse vascular-like networks under indirect coculture conditions all over the collagen scaffold imaged on day 21 of the sandwich assay. HUVECs are immuno-stained using CD31 antibody (green). Inset shows the presence of vascular-like networks on a different z-plane at a representative position inside the encircled area (scale bar 500 µm). Due to manual gel casting the resulting collagen layer for cell seeding is not perfectly uniform in thickness at the micrometer scale, thus the visualization of the whole scaffold cannot be accomplished within a single z-range for all the tiles. **(B)** 3D orthogonal projection of the formed network expanding over a maximum z-range of 600 µm.

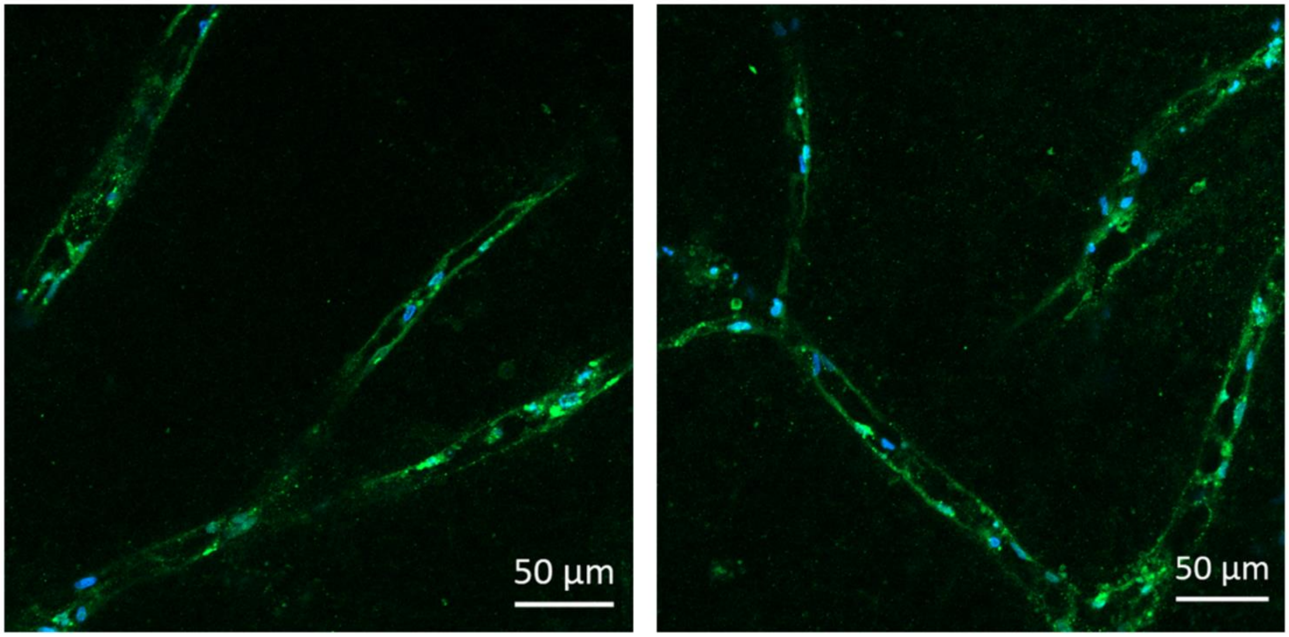

Supplementary Figure 3. Representative CLSM images of VE-cadherin immuno-stained vascular-like networks on day 21 showing the presence of VE-cadherin (green) on the endothelial surface. Nuclei were counterstained with Hoechst (blue).

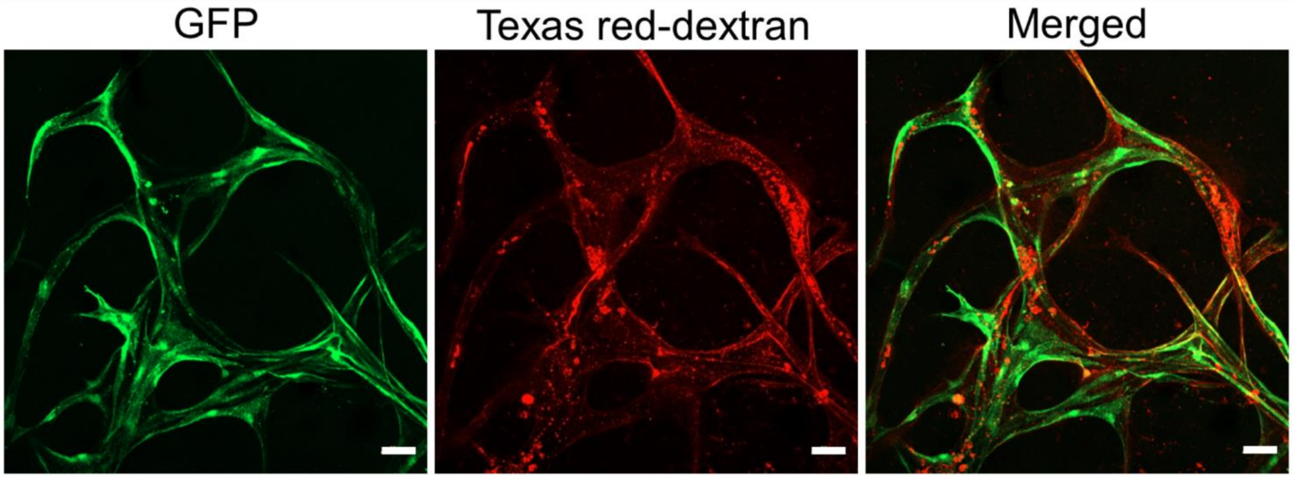

Supplementary Figure 4. CLSM images showing the permeability of GFP-HUVEC vascular-like networks (green) towards Texas red-dextran (TD, red) of 10 kDa molecular weight. Cells were treated with TD after day 21 of culture (scale bar 50 µm).

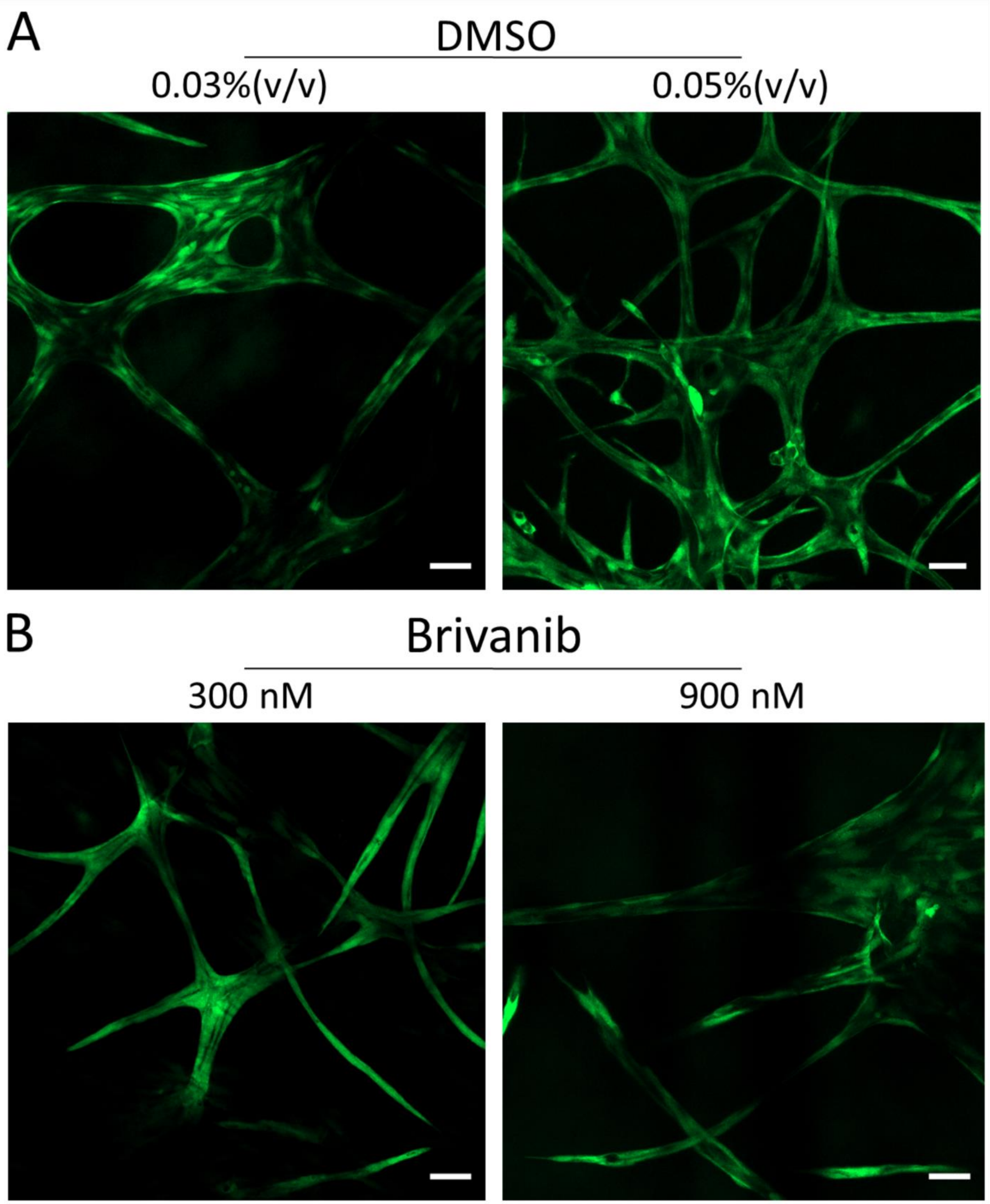

Supplementary Figure 5. Representative CLSM images showing **(A)** vascular-like networks formed under indirect coculture conditions imaged on day 14 when treated with DMSO starting on day 0 (vehicle controls for the respective analogous brivanib treatment); **(B)** Regeneration of the vascular-like networks imaged 14 days after aborting brivanib treatment on day 14. Cells in both experiments were stained with FDA (green) and PI (red) (scale bar 50 µm).

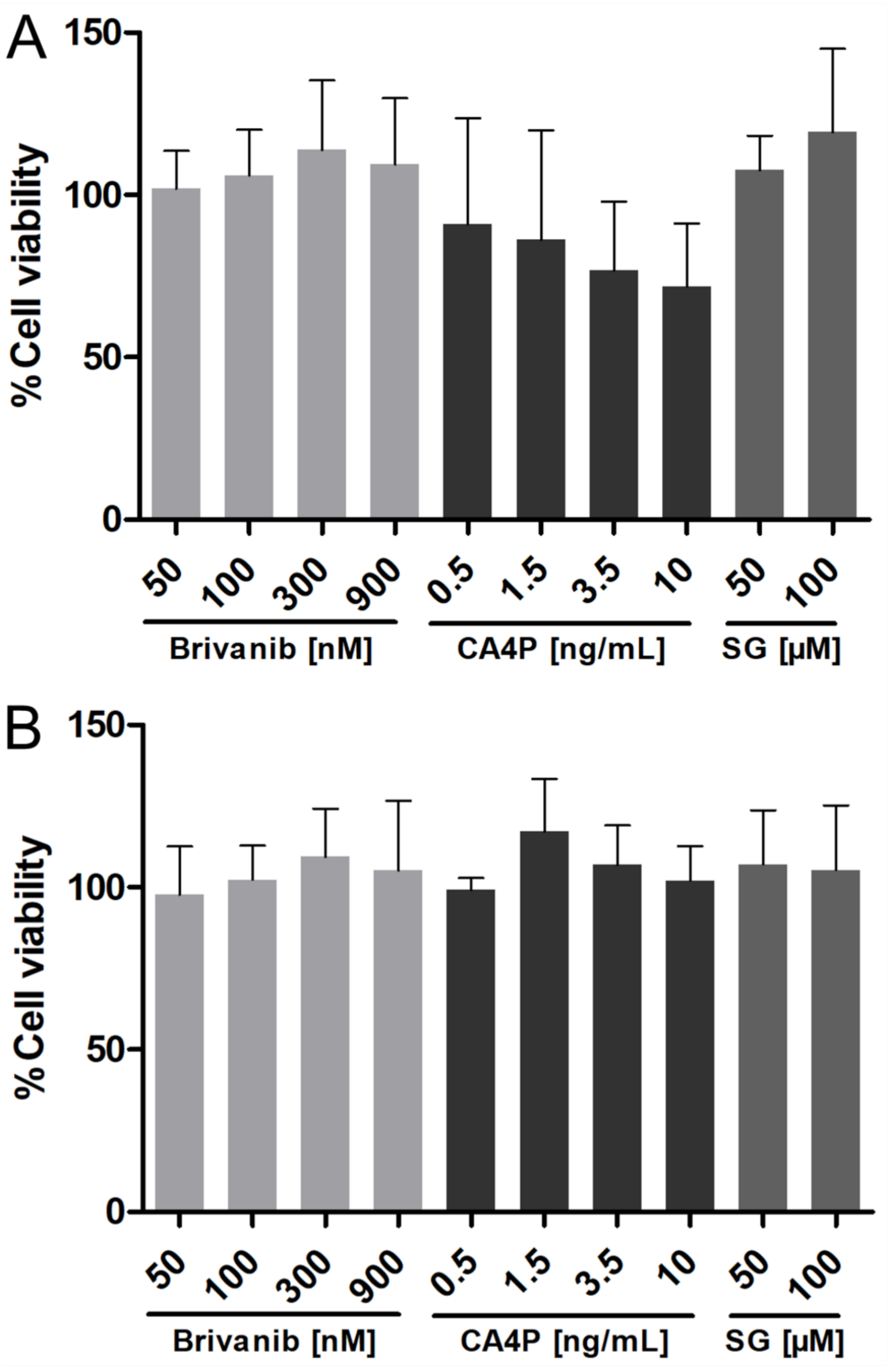

Supplementary Figure 6. MTS based cell viability of **(A)** HUVECs and **(B)** HDFs after treatment with the respective drug for 48 h. The viability was tested with a cell density similar to the indirect coculture. Data are shown as mean ± SD (n=4). Differences of all groups to control are non-significant ($p>0.01$, Mann-Whitney U-Test).

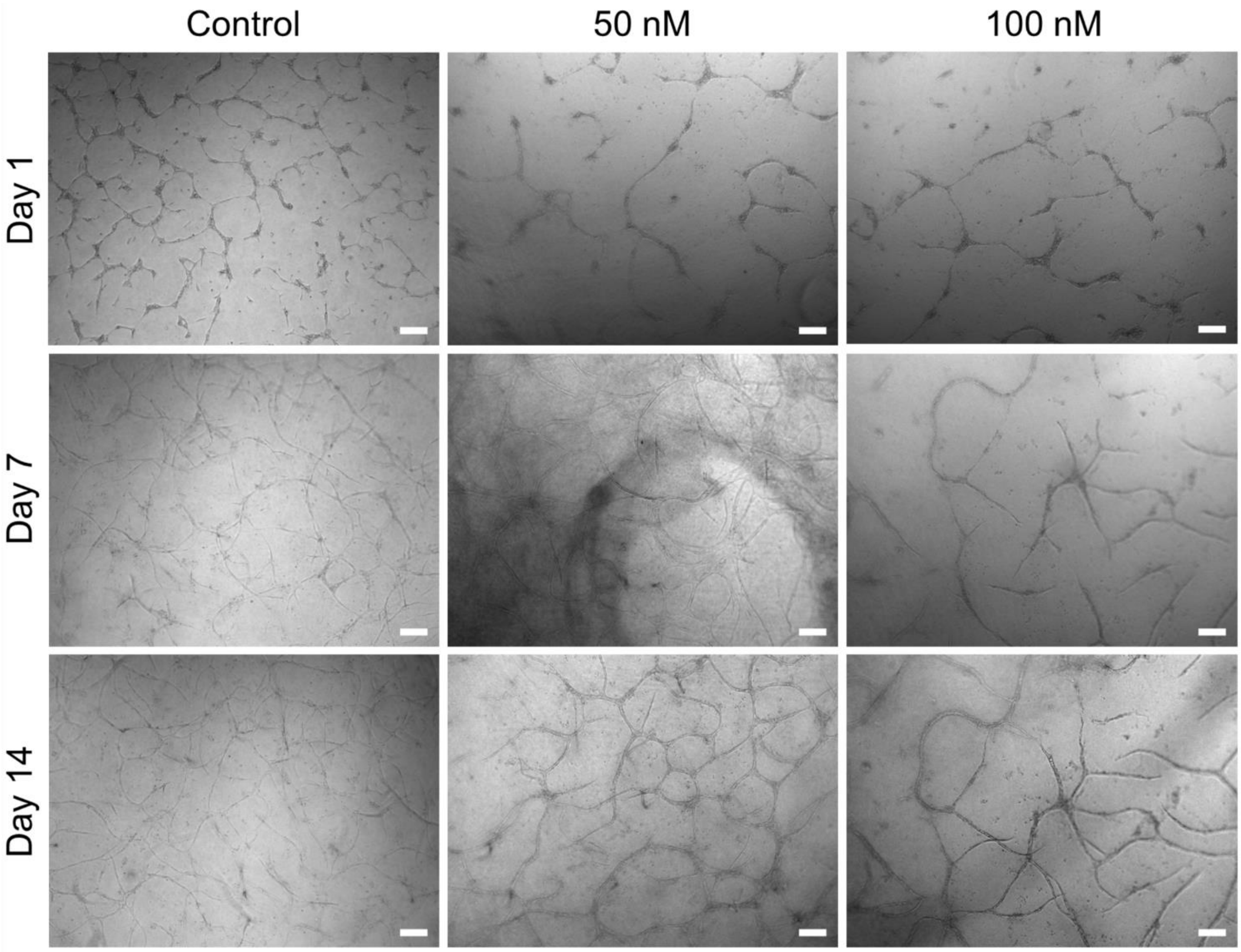

Supplementary Figure 7. Representative brightfield microscopy images of the vascular-like networks under concentration-dependent treatment with brivanib starting on day 0 of vascular network initiation in indirect coculture (scale bar 200 µm).

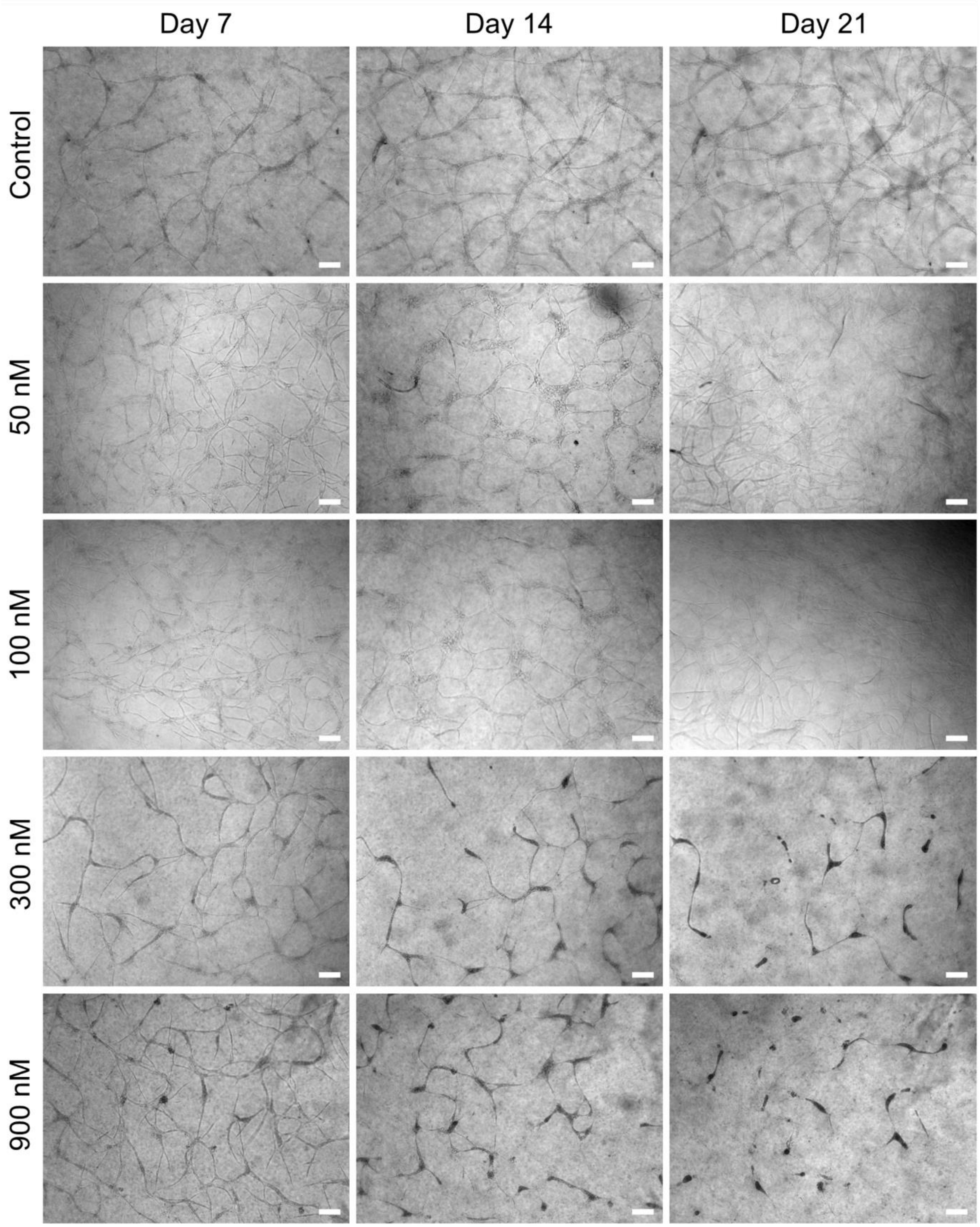

Supplementary Figure 8. Representative brightfield microscopy images of the vascular-like networks under concentration-dependent treatment with brivanib starting on day 7 after initiation of network formation in indirect coculture (scale bar 200 µm).

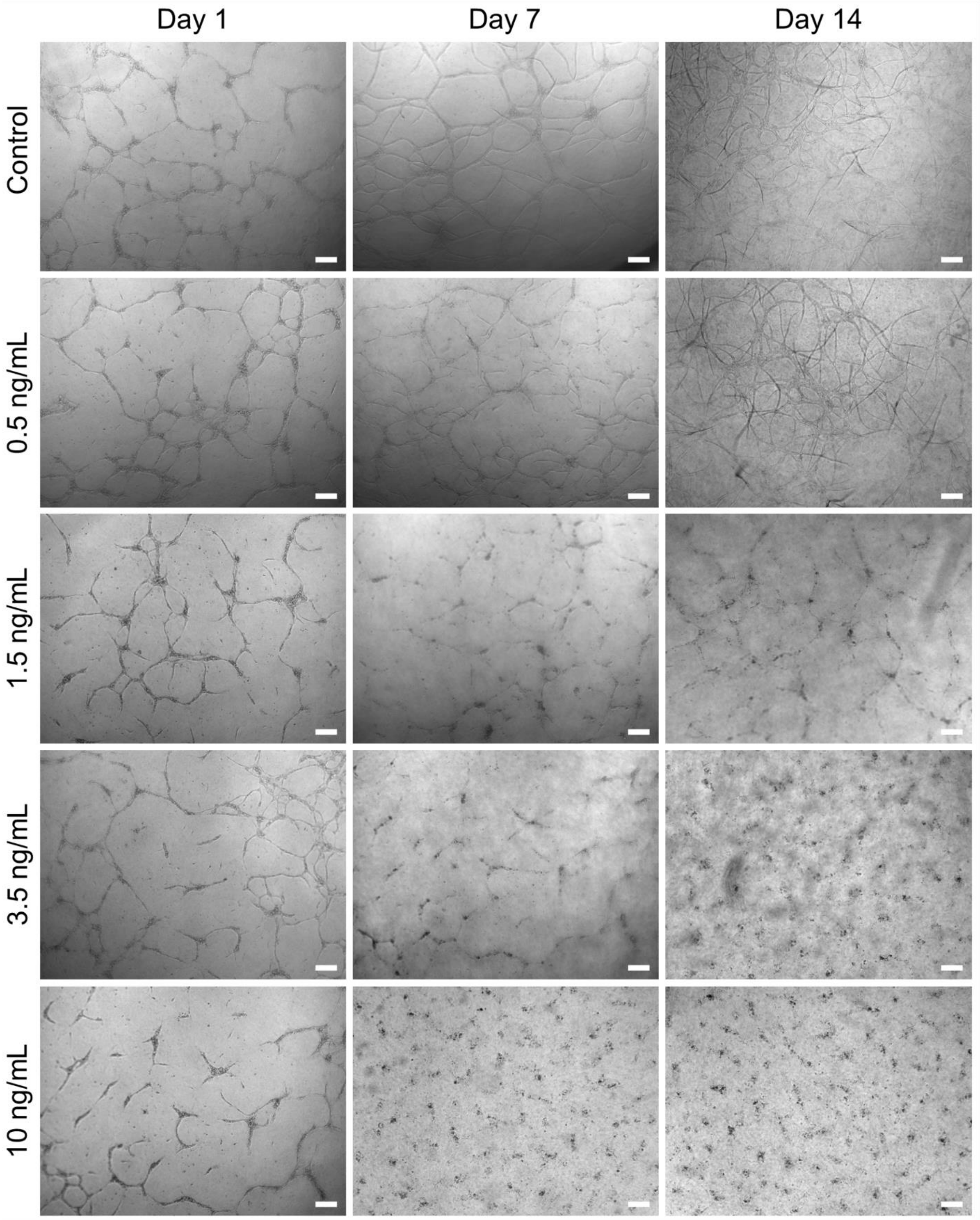

Supplementary Figure 9. Representative brightfield microscopy images of the vascular-like network under concentration-dependent treatment with combretastatin-A4-phosphate (CA4P) starting on day 0 after initiation of network formation in indirect coculture (scale bar 200 µm).

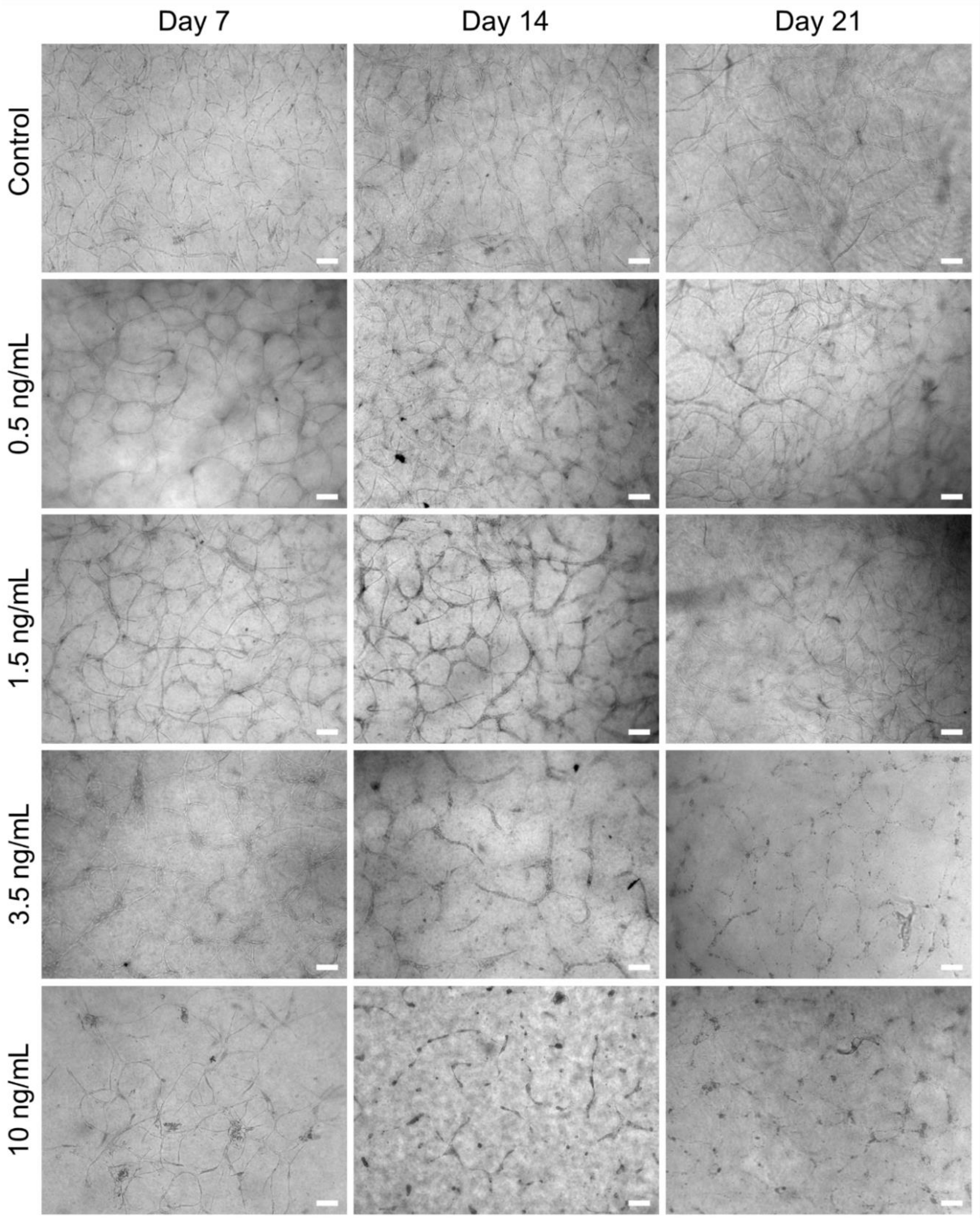

Supplementary Figure 10. Representative brightfield microscopy images of the vascular-like network under concentration-dependent treatment with combretastatin-A4-phosphate (CA4P) starting on day 7 after initiation of network formation in indirect coculture (scale bar 200 µm). Note, that a granulated background occurs in brightfield imaging when the network is located close to the PET membrane of the insert.

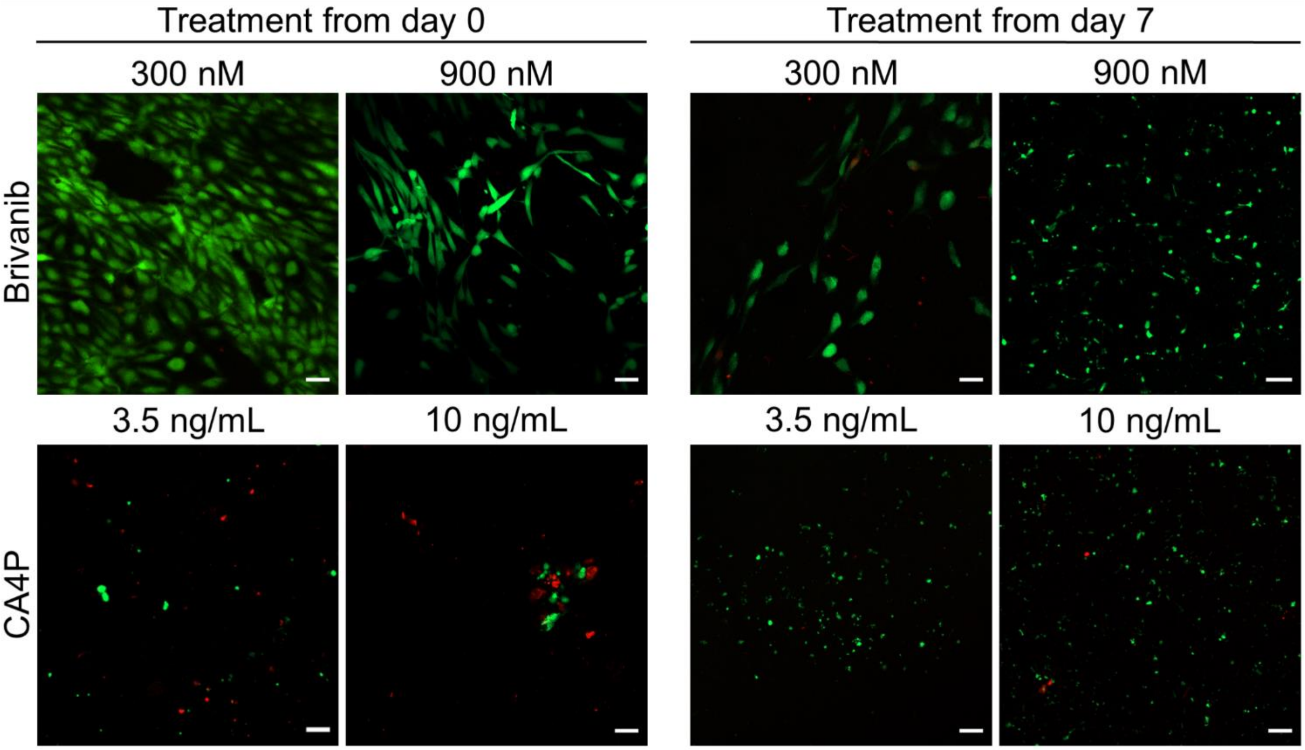

Supplementary Figure 11. Representative CLSM images of the live-dead stained HUVECs in indirect coculture (FDA green, PI red) after the concentration dependent treatment with brivanib or combretastatin-A4-phosphate (CA4P) starting on day 0 and 7. Upon drug treatment confluent layers of HUVECs were observed at the initial cell seeding plane when treatment started on day 0 in addition to disrupted networks when treatment started on day 7 (scale 50 µm).

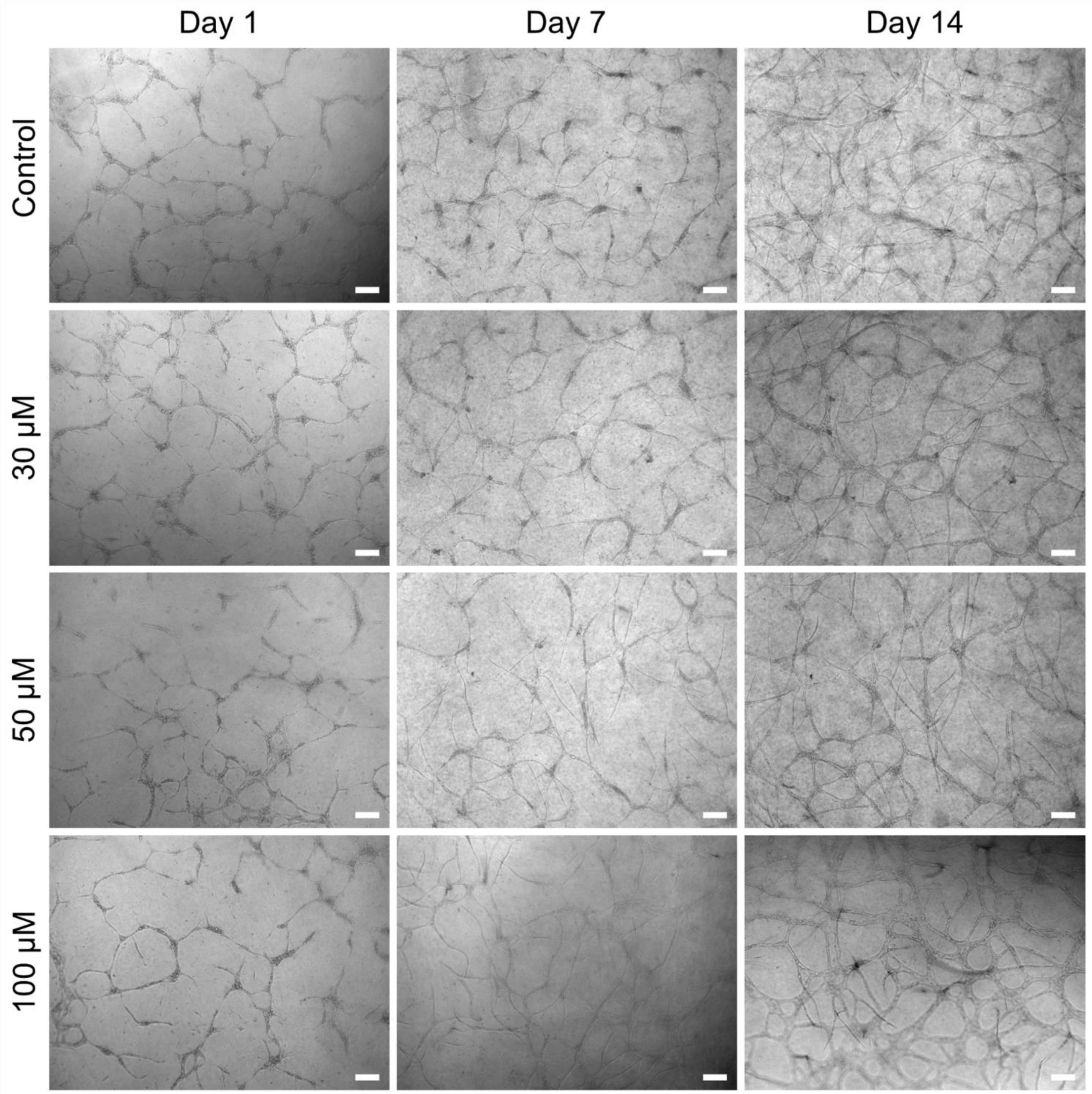

Supplementary Figure 12. Representative brightfield microscopy images of the vascular-like networks under concentration-dependent treatment with 6'-sialylgalactose (SG) starting on day 0 of induction of network formation in indirect coculture (scale bar 200 µm).

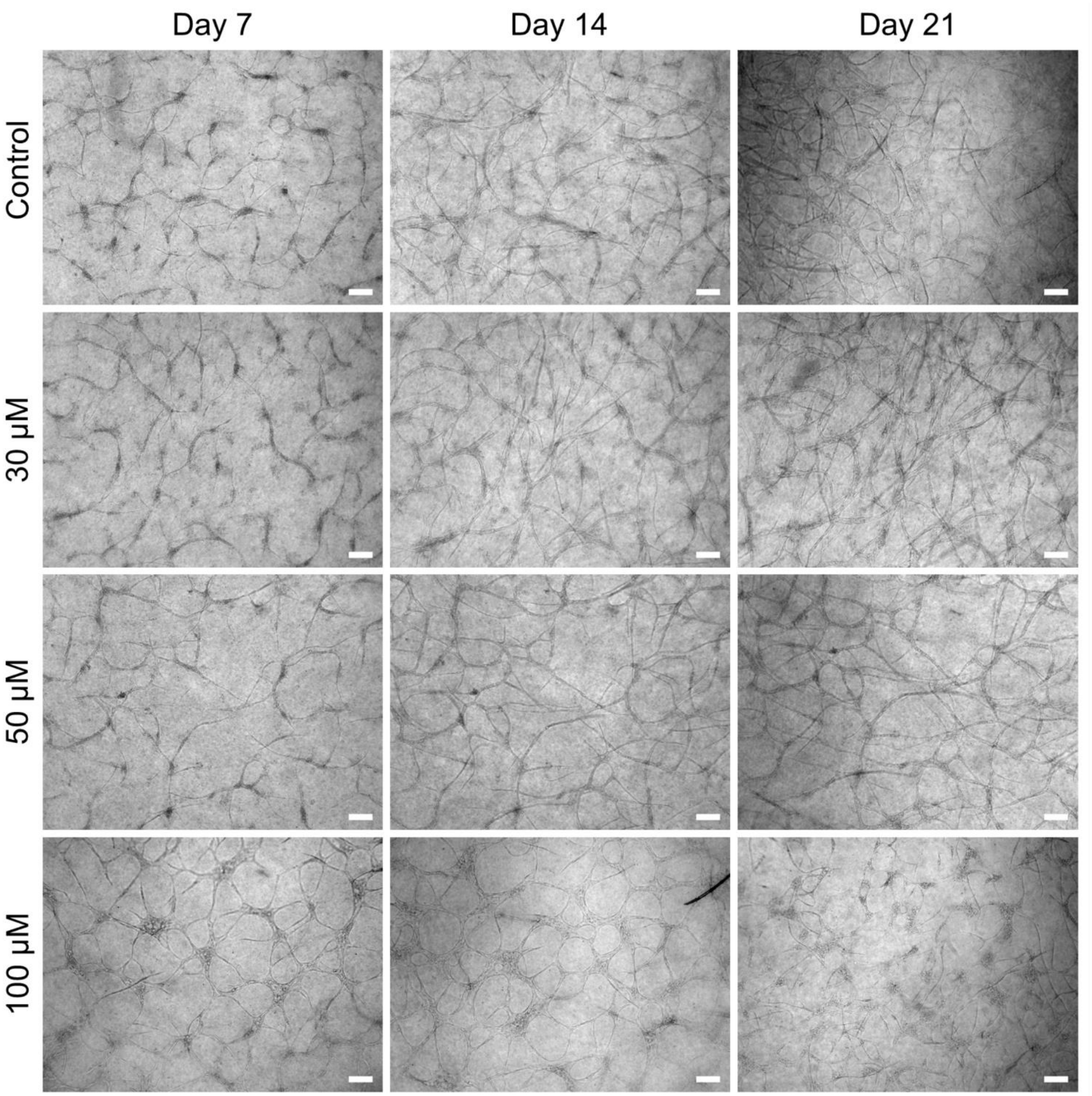

Supplementary Figure 13. Representative brightfield microscopy images of the vascular-like networks under concentration-dependent treatment with 6'-sialylgalactose (SG) starting on day 7 after initiation of vascular network formation in indirect coculture (scale bar 200 µm).

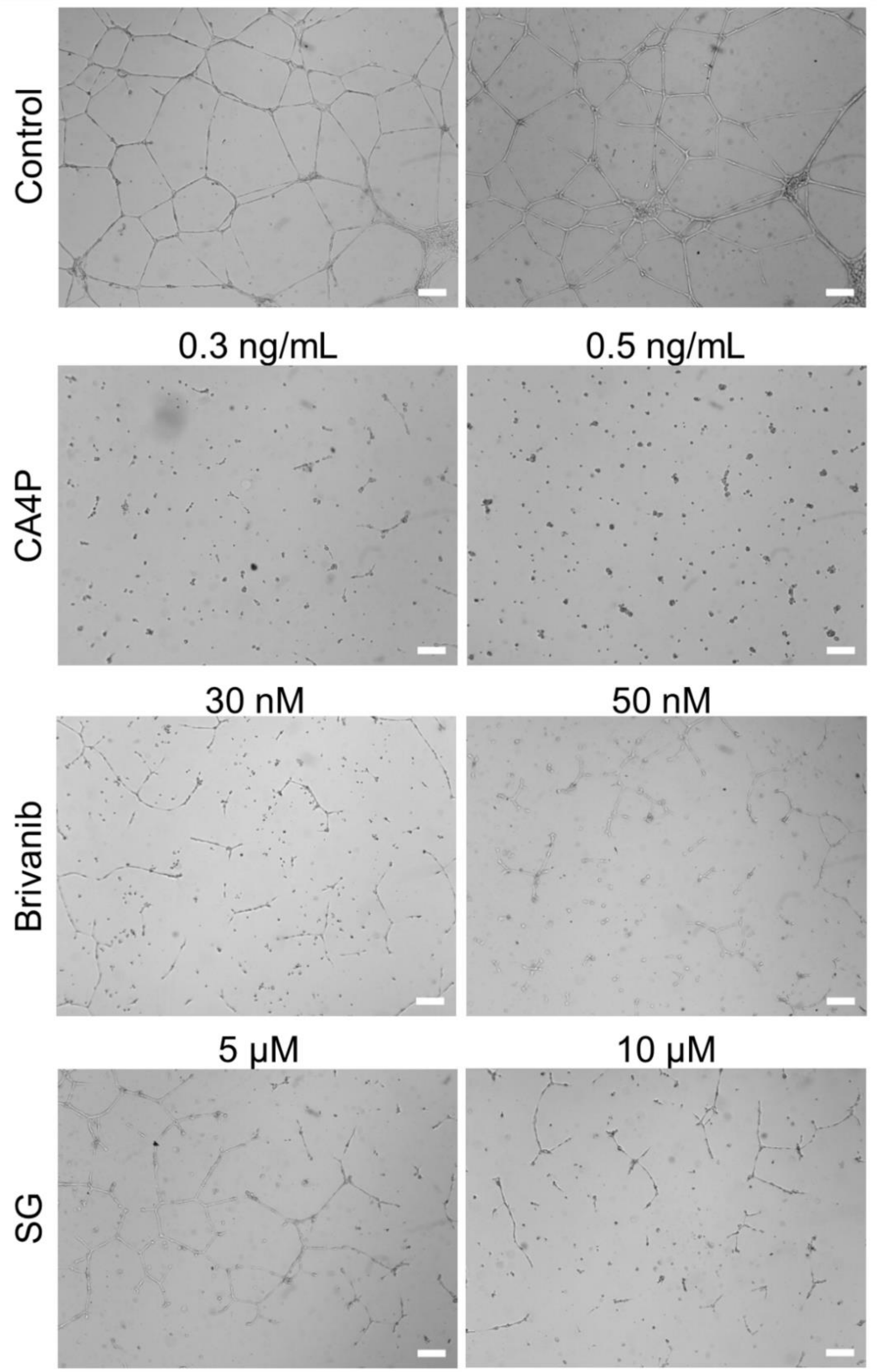

Supplementary Figure 14. Representative brightfield microscopy images showing the antiangiogenic effects of combretastatin-A4-phosphate (CA4P), brivanib and 6'-sialylgalactose (SG) tested using a conventional tube formation assay on Matrigel™ (24 h). The drugs were able to prevent angiogenesis at low concentrations of 0.3 ng·mL^{-1}, 50 nM and 10 µM, respectively (scale 200 µm).

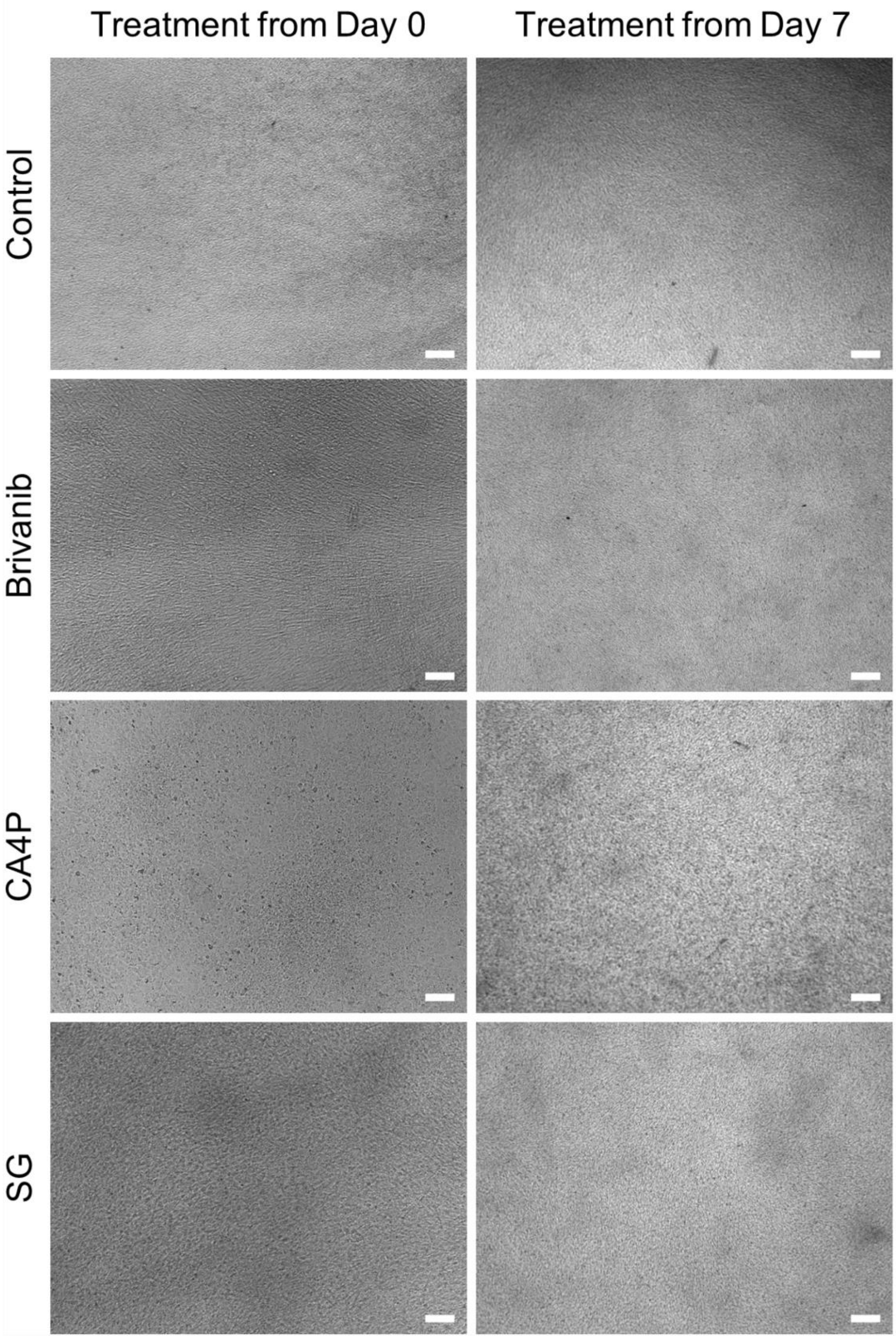

Supplementary Figure 15. Brightfield microscopy images of confluent HDF monolayers in the bottom compartment of the indirect cocultures after drug treatment with brivanib (900 nM), combretastatin-A4-phosphate (CA4P, 10 ng·mL^{-1}) and 6'-sialylgalactose (SG, 100 µM) on day 14 and day 21 respectively. Confluent HDF layers with no obvious sign of cytotoxicity indicated by detaching dead cells were observed (scale bar 200 µm).

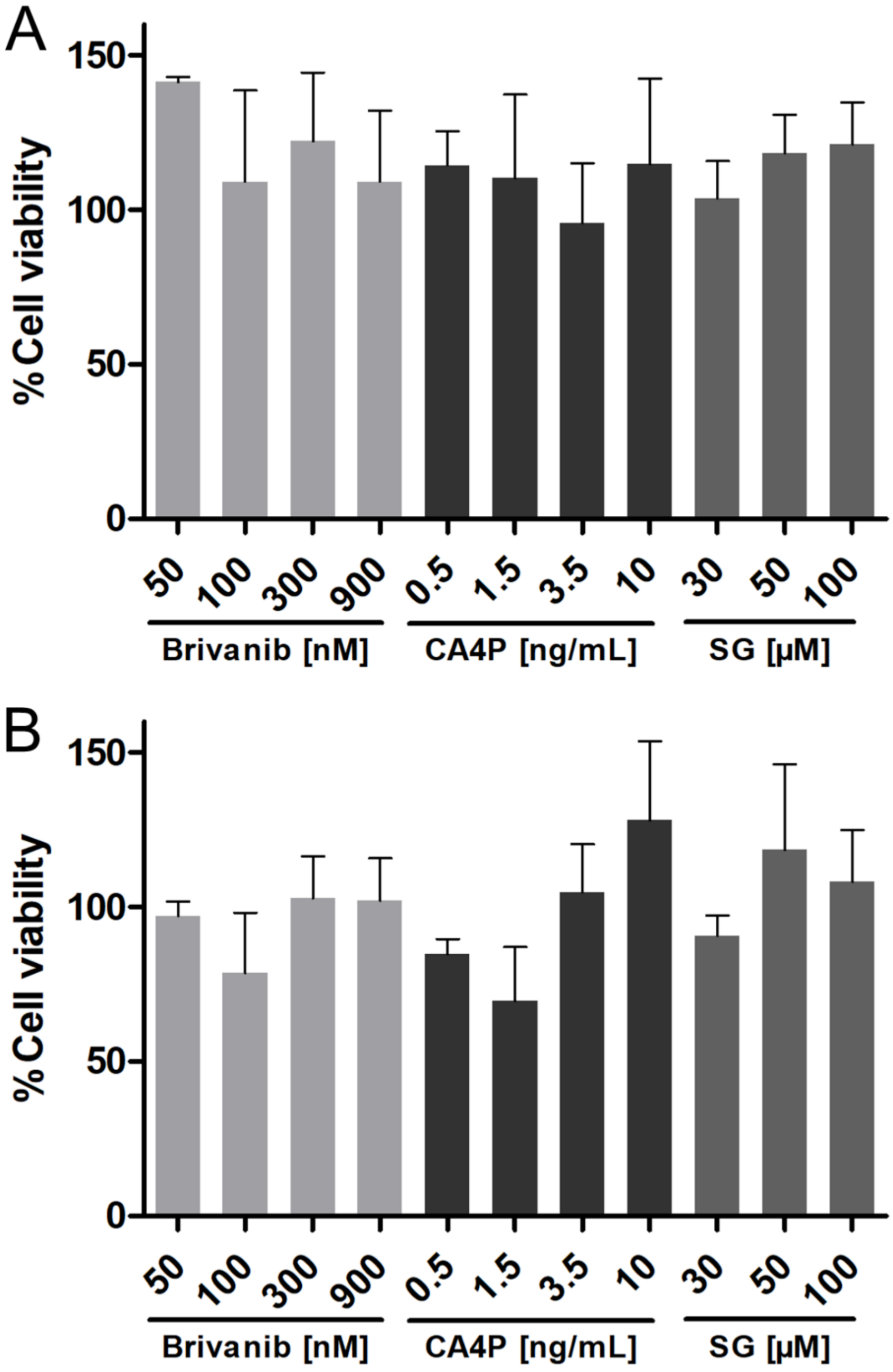

Supplementary Figure 16. Cell viability of confluent HDFs in the indirect co-culture post drug treatment **(A)** on day 14 for the drug treatment starting from day 0 and **(B)** on day 21 for the drug treatment starting from day 7 onwards. Data are shown as mean ± SD (n=2-6). Differences of all groups to control are non-significant ($p>0.01$, Mann-Whitney U-Test).
